# Deconstructive Synthesis of Bridged and Fused Rings via Transition-Metal-Catalyzed “Cut-and-Sew” Reactions of Benzocyclobutenones and Cyclobutanones

**DOI:** 10.1021/acs.accounts.2c00400

**Published:** 2022-07-28

**Authors:** Yibin Xue, Guangbin Dong

**Affiliations:** † Department of Chemistry, University of Chicago, Chicago, Illinois 60637, United States

## Abstract

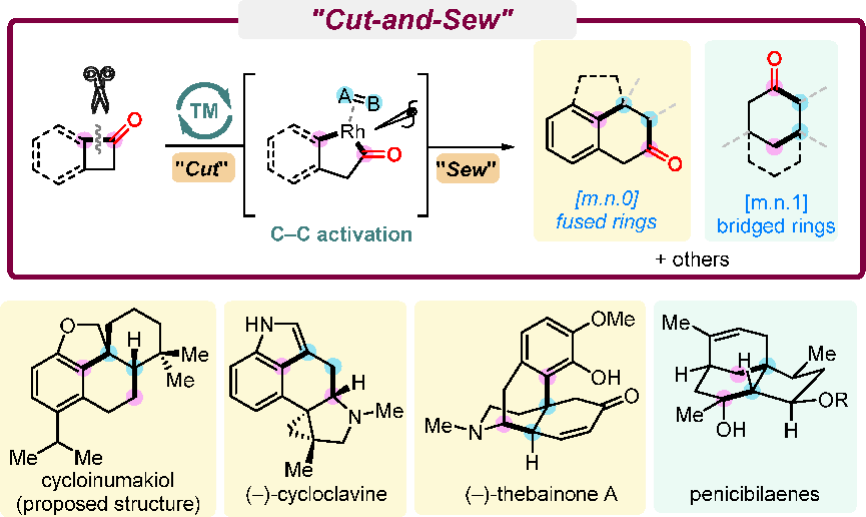

Bridged and fused rings are commonly found in biologically important molecules. Current tactics to construct these ring systems are primarily based on stepwise ring formation (i.e., making one ring first followed by making another) and cycloaddition reactions (e.g., Diels–Alder reaction). To seek a complementary and perhaps more unified ring-forming approach, a deconstructive strategy based on C–C bond activation of cyclic ketones has been conceived. The named “cut-and-sew” reaction uses cyclic ketones with a tethered unsaturated moiety as substrates, which involves oxidative addition of a transition metal into the ketone C–C bond followed by intramolecular insertion of the unsaturated unit. This strategy has proved successful to access diverse ring scaffolds that are nontrivial to construct otherwise.

This Account offers a concise summary of our laboratory’s systematic efforts in developing transition metal-catalyzed cut-and-sew reactions for the synthesis of bridged and fused rings over the past 10 years. In particular, we will focus on the reactions using readily available benzocyclobutenones and cyclobutanones. To date, the scope of the cut-and-sew reactions has been greatly expanded. First, diverse unsaturated moieties can serve as suitable coupling partners, such as alkenyl, alkynyl, allenyl, carbonyl, and iminyl groups. Second, a variety of reaction modes have been uncovered. In this account, (4 + 2), (4 + 2 – 1), and (4 + 1) cycloadditions that lead to a range of bridged or fused scaffolds will be summarized. Third, enantioselective transformations have been realized to efficiently construct chiral scaffolds, which are enabled by two strategies: enantio-determining migratory insertion and desymmetrization of cyclobutanones. Fourth, the synthetic applications have been demonstrated in streamlined total syntheses of a number of complex natural products. Compared to conventional synthetic logics, the cut-and-sew reaction allows the development of new bond-disconnecting strategies. Thus, the syntheses of (−)-cycloclavine, (−)-thebainone A, penicibilaenes, and the proposed cycloinumakiol are discussed in more detail.

In addition to the narrative of the development of the cut-and-sew chemistry, this Account also aims to provide core guiding foundations and inspirations toward broader deconstructive synthetic applications through C–C bond cleavage. It is anticipated that more classes of cyclic compounds could serve as the substrates beyond benzocyclobutenones and cyclobutanones, and more diverse unsaturated moieties could be coupled. It can also be envisaged that more innovative utilization of this cut-and-sew strategy in complex organic syntheses will be revealed in the near future.

## Key References

- Xu, T.; Dong, G. Rhodium-Catalyzed Regioselective Carboacylation of Olefins: A C–C Bond Activation Approach for Accessing Fused-Ring Systems. *Angew. Chem., Int. Ed*. **2012**, *51*, 7567–7571.^1^*The concept of the cut-and-sew transformations to construct fused rings is illustrated.*
- Ko, H.; Dong, G. Cooperative Activation of Cyclobutanones and Olefins Leads to Bridged Ring Systems by a Catalytic [4 + 2] Coupling. *Nat. Chem.***2014**, *6*, 739–744.^2^*The challenge of using cyclobutanones in the cut-and-sew reaction is addressed by adding a temporary directing group.*
- Deng, L.; Chen, M.; Dong, G. Concise Synthesis of (−)-Cycloclavine and (−)-5-*epi*-Cycloclavine via Asymmetric C–C Activation. *J. Am. Chem. Soc.***2018**, *140*, 9652–9658.^3^*The potential of the cut-and-sew reaction is demonstrated in the rapid and enantioselective construction of multiple fused rings in indole alkaloids.*
- Xue, Y.; Dong, G. Total Synthesis of Penicibilaenes via C–C Activation-Enabled Skeleton Deconstruction and Desaturation Relay-Mediated C–H Functionalization. *J. Am. Chem. Soc.***2021**, *143*, 8272–8277.^4^*The “C–C/C–H” two-stage strategy is conceived and demonstrated in concise total synthesis.*

## 1 Introduction

With an increasing demand to escape from flatland,^5^ synthetic methods that can efficiently generate novel, complex, sp^3^-rich structures, such as bridged and fused rings, become more and more attractive to medicinal chemists. Conventionally, bridged and fused rings were often prepared via either stepwise ring formation or cycloaddition. The stepwise approaches involve making one ring first and then another; thus, they generally require multistep operations, along with some functional group manipulations and protecting group usages. On the other hand, cycloaddition reactions, such as the Diels–Alder reaction, have been highly powerful in constructing various ring systems and successfully demonstrated in numerous elegant total syntheses.^6^ Typically, different classes of substrates are required in order to access different ring systems, and certain ring structures, such as those relying on forming “anti-Bredt” intermediates, are more challenging to construct with cycloaddition-based strategies.

**Scheme 1:**
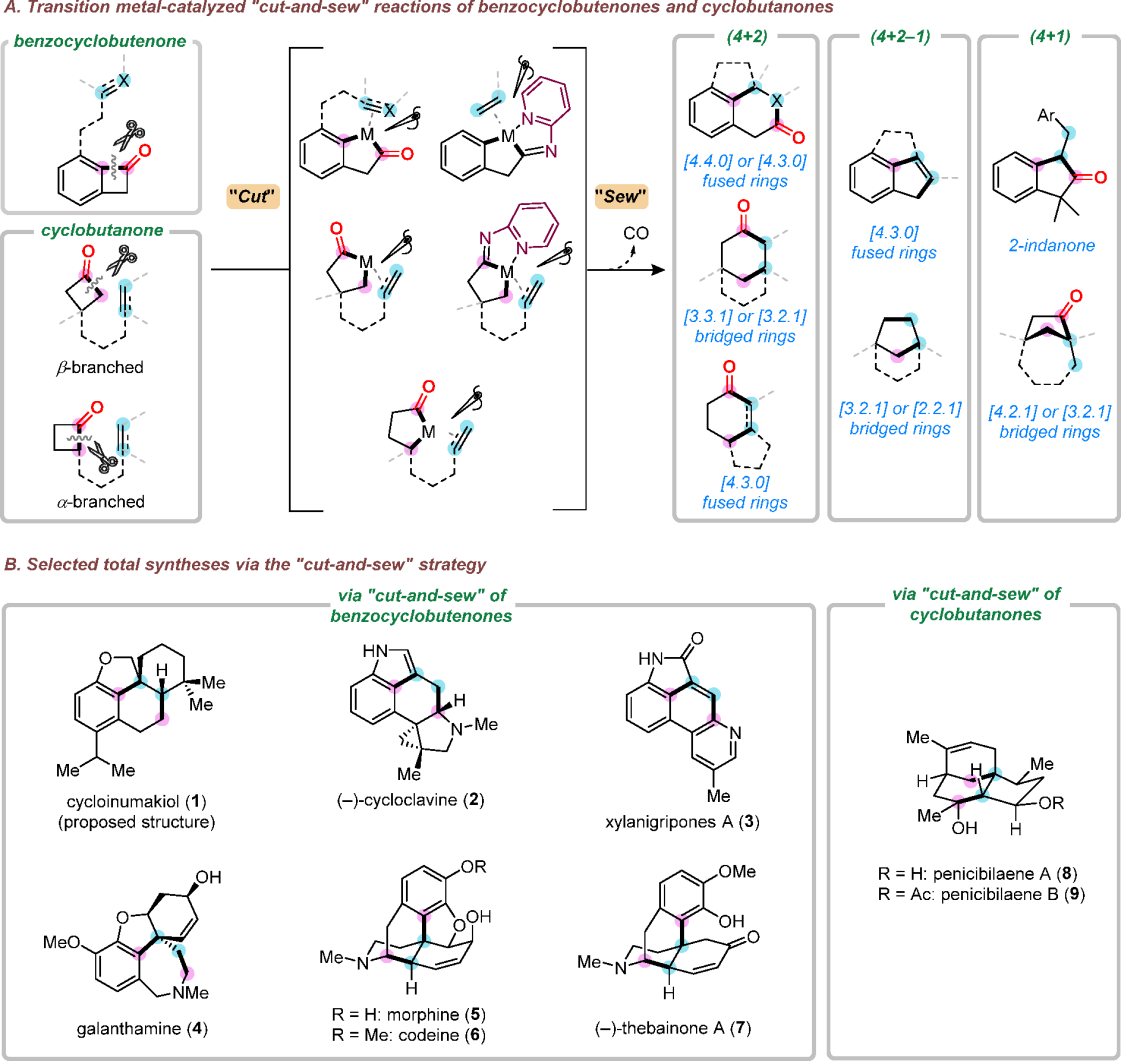
Cut-and-Sew Reactions of Benzocyclobutenones and Cyclobutanones

Transition metal (TM)-catalyzed carbon–carbon (C–C) bond activation has emerged as a useful tool for devising unusual bond-disconnecting strategies.^7^ To seek a complementary ring-forming approach, a deconstructive strategy based on C–C bond activation of readily available cyclic ketones has been conceived (Scheme 1A). This named “cut-and-sew” strategy^8^ uses cyclic ketones with a tethered unsaturated moiety as substrates. It starts with the oxidative addition of a TM into the ketone C–C bond (the “cut” step) to give a reactive metallacycle, followed by intramolecular migratory insertion of the unsaturated unit and reductive elimination to furnish the ring (the “sew” step). It has been hypothesized that, by changing the ring sizes of the cyclic ketones, the length of the linkers, and different unsaturated coupling partners, diverse bridged and fused ring scaffolds would be constructed by this unified method. In addition, considering that the carbonyl moiety in ketones can be extruded under certain conditions during the C–C activation processes, the decarbonylative cut-and-sew reactions (with CO deletion) can also be realized with ketone-based substrates. Moreover, apart from the normal 2π insertion, TM-catalyzed one-carbon ring expansions have also been achieved to generate intriguing products.

On the basis of the rich prior knowledge of C–C activation,^7,9^ our group has been exploring the TM-catalyzed cut-and-sew reactions and their applications in complex molecule synthesis since 2012.^1^ In the past 10 years, benzocyclobutenones and cyclobutanones have been the two main classes of substrates we have studied. To date, several different types of cut-and-sew reactions with benzocyclobutenones and cyclobutanones have been developed in our laboratory, including (a) (4 + 2) or (4 + 2 – 1) cycloaddition between benzocyclobutenones and 2π units to construct [m.n.0] fused rings; (b) (4 + 1) cycloaddition between benzocyclobutenones and stryenes to construct 2-indanones; (c) (4 + 2) cycloaddition between α-branched cyclobutanones and 2π units to construct [m.n.0] fused rings; and (d) (4 + 2), (4 + 2 – 1), or (4 + 1) cycloaddition between β-branched cyclobutanones and 2π units to construct [m.n.1] bridged rings. To illustrate how these ring-forming methods can facilitate syntheses of complex molecules, these C–C activation methods have been applied to a number of concise total syntheses (Scheme 1B).^3,4,10^ In this Account, we first summarize our development of these catalytic cut-and-sew methods with benzocyclobutenones and cyclobutanones, followed by discussions of streamlined syntheses of (−)-cycloclavine, (−)-thebainone A, penicibilaenes, and the proposed cycloinumakiol, as representative examples enabled by such a deconstructive strategy.

## 2 Development of the Cut-and-Sew Methods

### 2.1 (4 + 2) Cut-and-Sew Reactions of Benzocyclobutenones

**Scheme 2:**
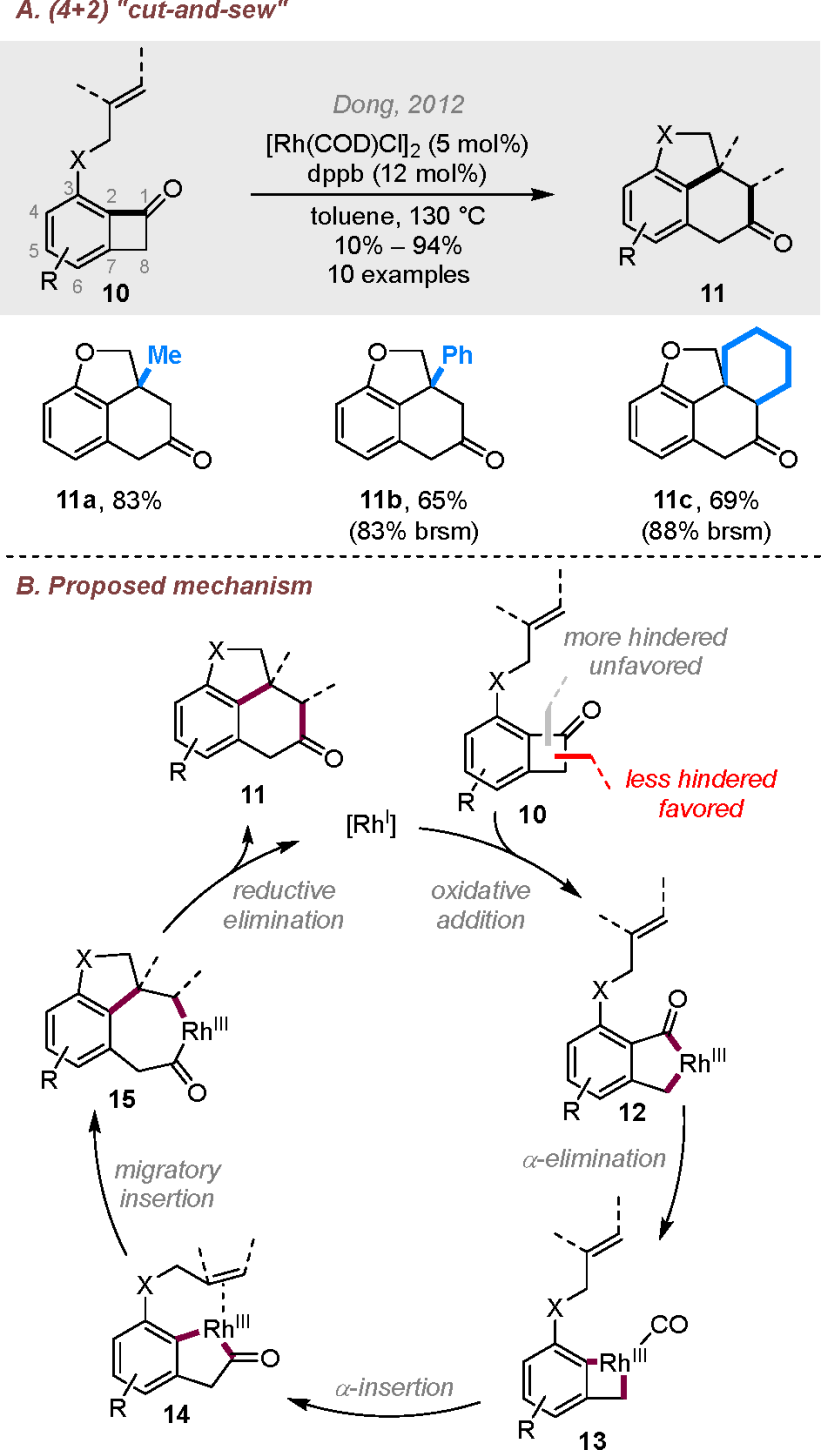
Rhodium-Catalyzed Intramolecular (4 + 2) Reactions between Benzocyclobutenones and Alkenyl Groups

**Scheme 3:**
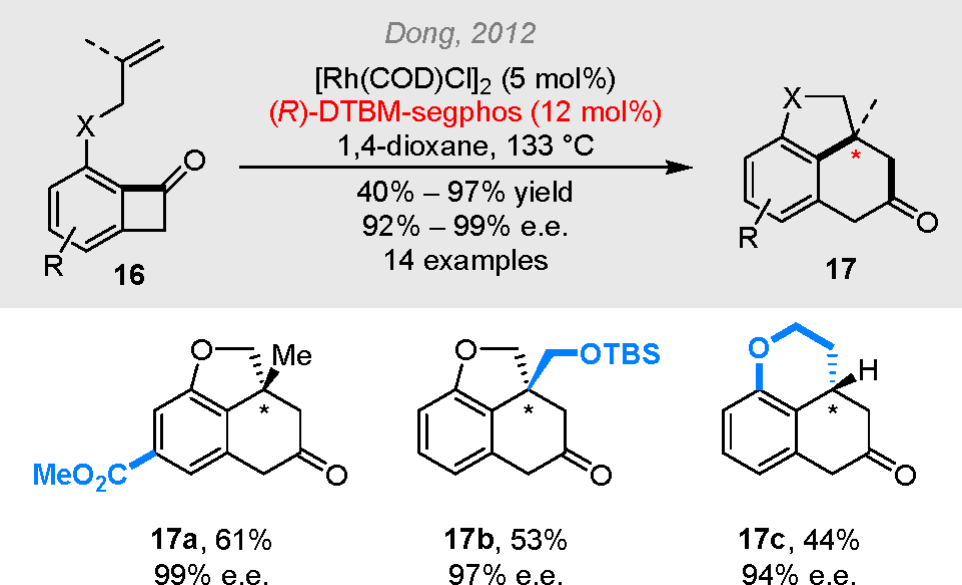
Rhodium-Catalyzed Asymmetric Cut-and-Sew Reactions between Benzocyclobutenones and Alkenyl Groups

Benzocyclobutenones are a common class of four-membered ring ketones.^11,12^ They can be easily accessed by diverse methods, including [2 + 2] cycloaddition with benzynes,^12b,12c,12e,12g^ intramolecular nucleophilic addition,^12a^ transition metal-catalyzed intramolecular C–H functionalization,^12d,12f^ and photoinduced cyclization.^12h^ Driven by strain release (their ring strain is higher than saturated cyclobutanones),^13^ benzocyclobutenones are excellent substrates for transition-metal-mediated C–C bond activation. Inspired by Liebeskind et al.’s seminal organometallic studies,^9a,9c^ we reported the first Rh-catalyzed intramolecular cut-and-sew reaction between benzocyclobutenones and alkenyl groups in 2012 (Scheme 2A).^1^ Note that the corresponding substrates based on parent cyclobutenones or those fused to nonbenzene aromatics are more difficult to prepare. The cut-and-sew reaction exhibited good functional group tolerance and worked for monosubstituted, 1,1- and 1,2-disubstituted, and trisubstituted alkenyl groups. Thus, it provides rapid access to benzo-fused tricyclic and tetracyclic rings. Later, detailed computational and experimental mechanistic studies showed that the reaction goes through a “rhodium migration” mechanism (Scheme 2B).^14^ The most favorable reaction path involves oxidative addition into the C(alkyl)–C(carbonyl) bond to generate intermediate **12**, followed by decarbonylation and CO-reinsertion to deliver rhodacycle **14**. Subsequent 2π-insertion and C–C reductive elimination provide the cut-and-sew product **11**. The enantioselective version of the reaction was also reported in 2012, in which DTBM-segphos was found to be a superior ligand to give up to 99% e.e. (Scheme 3).^15^

**Scheme 4:**
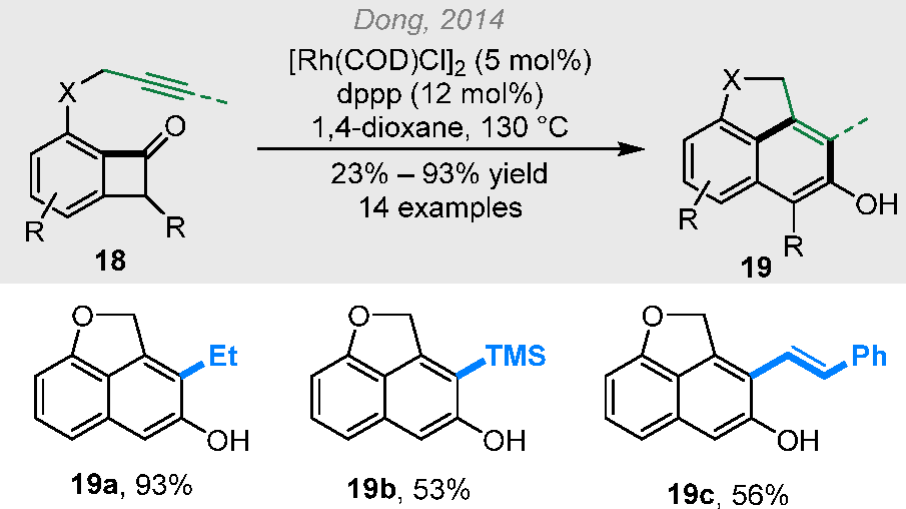
Rhodium-Catalyzed (4 + 2) Reactions between Benzocyclobutenones and Alkynyl Groups

**Scheme 5:**
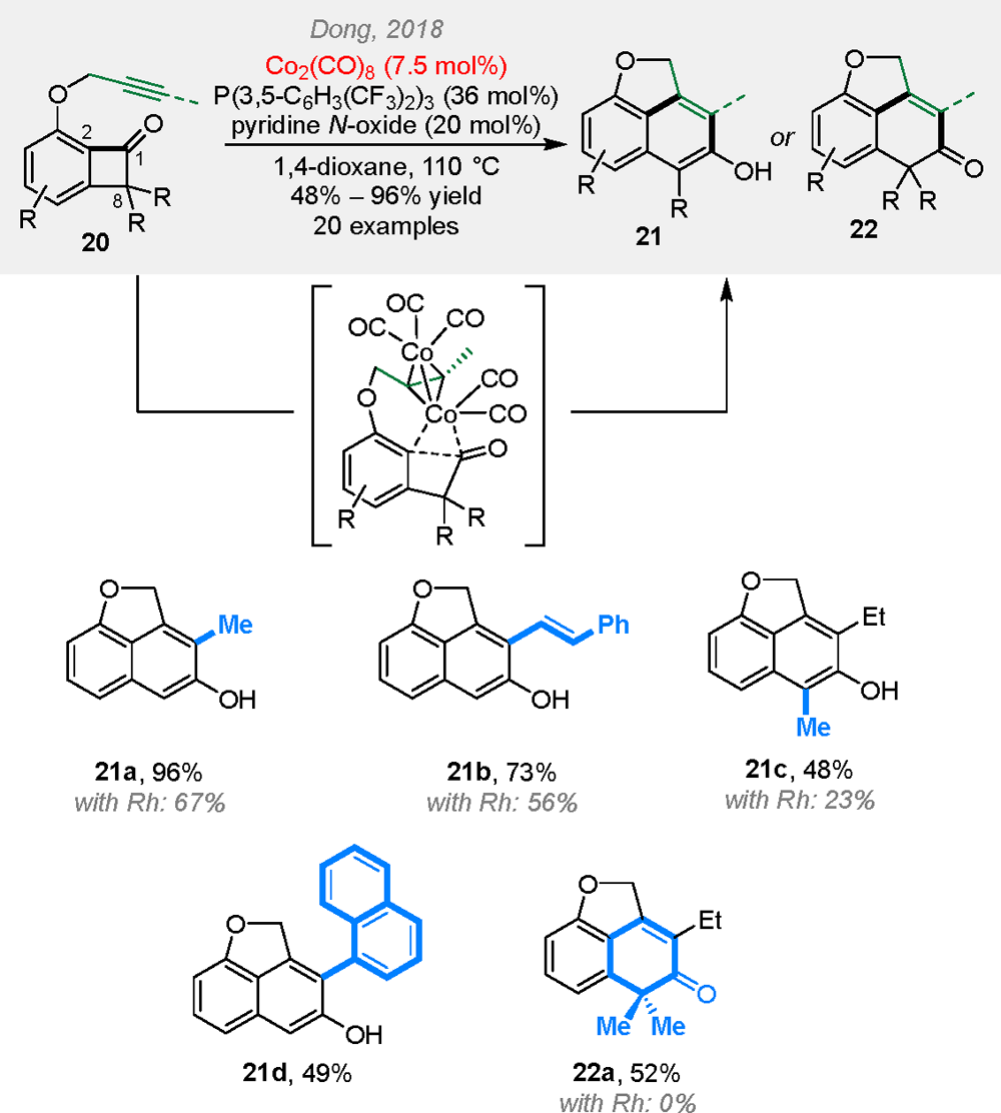
Cobalt-Catalyzed (4 + 2) Reactions between Benzocyclobutenones and Alkynyl Groups

Besides alkenyl groups, alkynyl groups were also found to be a suitable coupling partner in the cut-and-sew reaction with benzocyclobutenones (Scheme 4).^16^ With the C5-unsubstituted or monosubstituted substrates, the resulting (4 + 2) products underwent simultaneous aromatization to form 2-naphthols. In 2018, the same type of reactions was found to be catalyzed by an inexpensive cobalt complex (Scheme 5).^17^ Comparing to the corresponding Rh catalysis, the cobalt condition not only gives higher yields for some substrates but also allows C8-disubstituted benzocyclobutenones to couple, which were unreactive under the Rh conditions. A combined experimental and computational study shows that the reaction first forms a tetrahedral dicobalt–alkyne complex, which then undergoes oxidative addition into the C1–C2 bond with Co(0), followed by 2π insertion and reductive elimination.

**Scheme 6:**
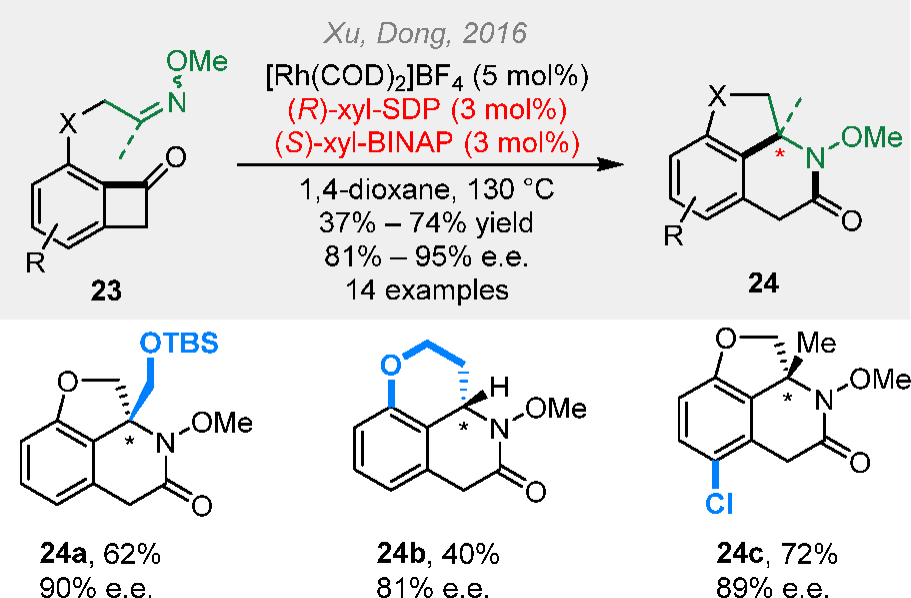
Rhodium-Catalyzed Asymmetric (4 + 2) Reactions between Benzocyclobutenones and Oxime Ethers

**Scheme 7:**
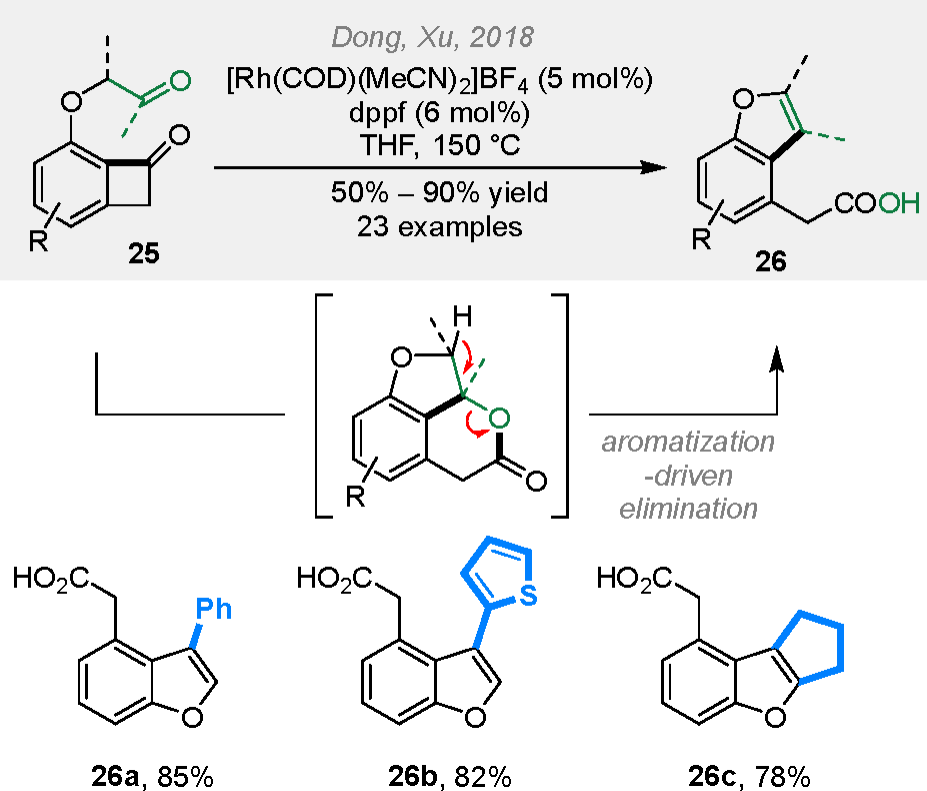
Rhodium-Catalyzed Cut-and-Sew Reactions between Benzocyclobutenones and Ketones/Aldehydes

Apart from alkenyl and alkynyl groups, more polar carbon–heteroatom double bonds can also serve as the coupling partners in the cut-and-sew reactions. In 2016, we reported an asymmetric cut-and-sew reaction between benzocyclobutenones and oxime ethers (Scheme 6).^18^ The combination of two chiral ligands delivered both high yield and high enantioselectivity of the chiral lactam products. The *N*-OMe group can be easily removed to give free lactams. Analogously, ketones and aldehydes can also serve as the 2π coupling partners (Scheme 7).^19^ In this case, after generation of the initial (4 + 2) cycloaddition product, the lactone ring is spontaneously opened via elimination to form a more stable benzofuran product (**26**).

### 2.2 (4 + 2) Cut-and-Sew Reactions with Cyclobutanones

**Scheme 8:**
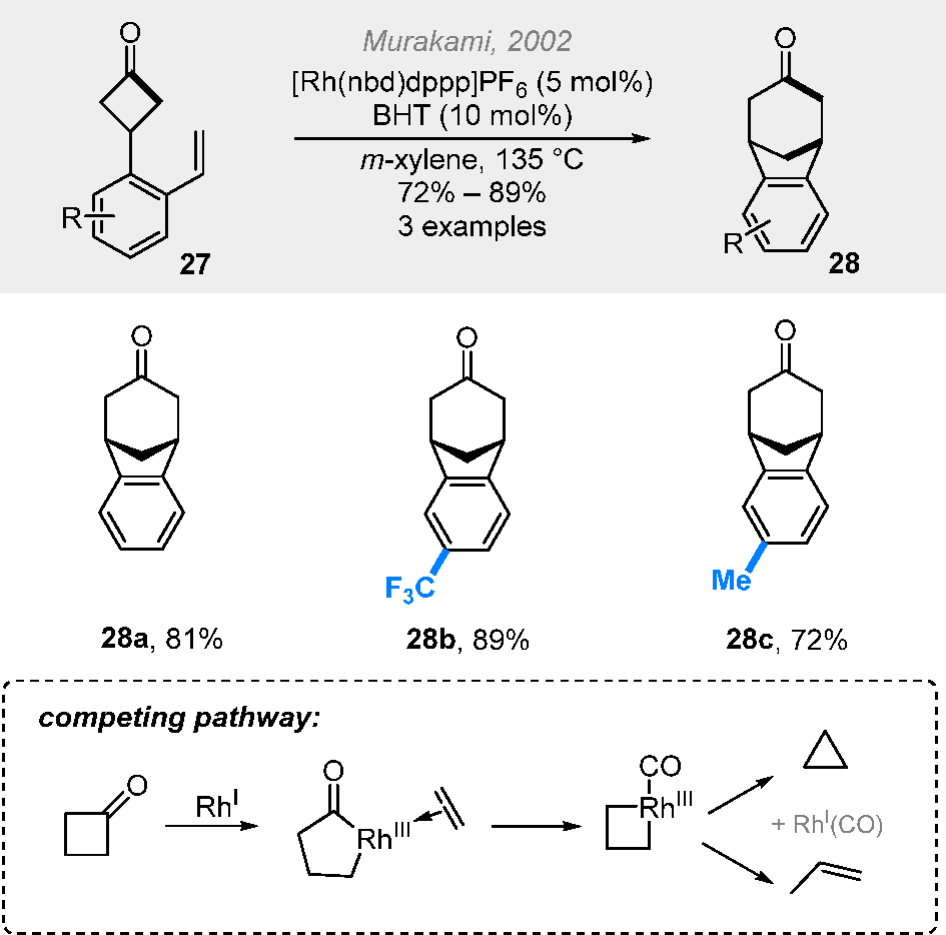
Rhodium-Catalyzed Intramolecular (4 + 2) Reactions with Benzene Linkers

**Scheme 9:**
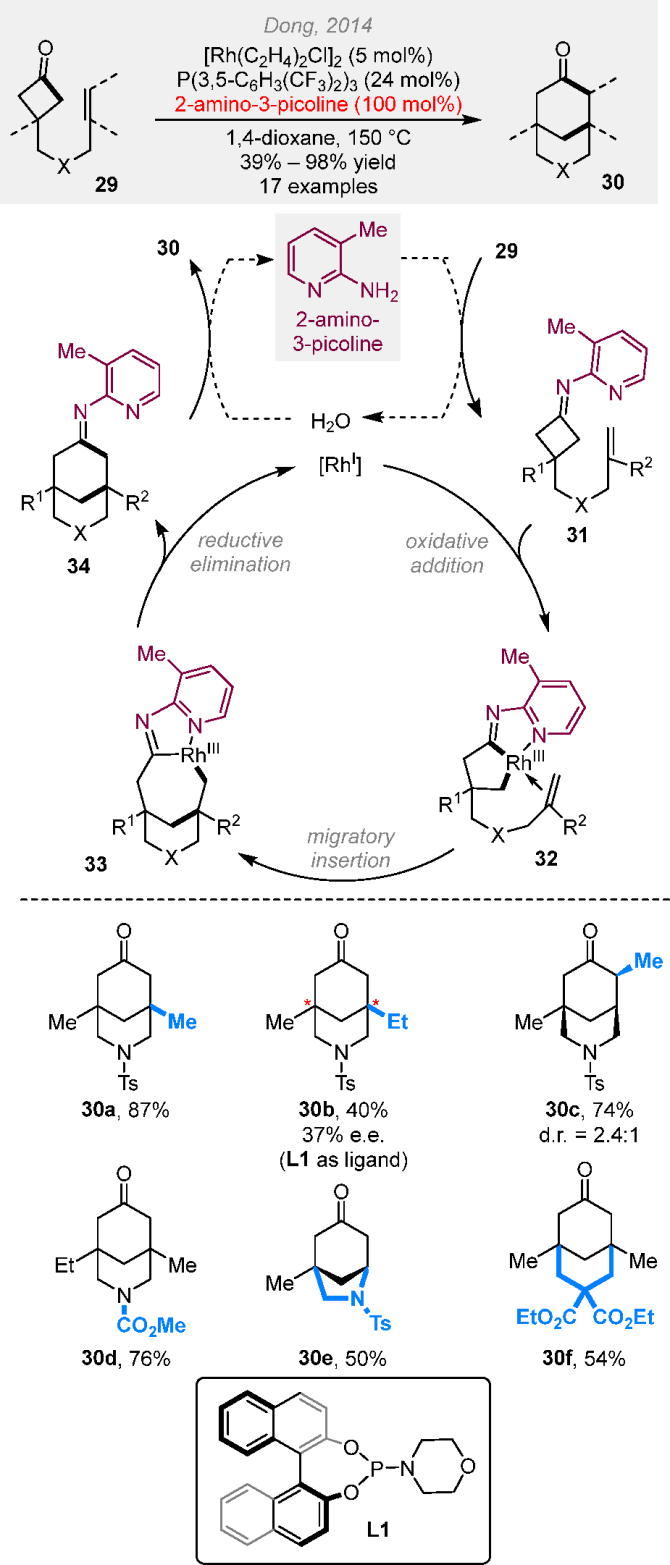
Temporary Directing Group-Enabled Cut-and-Sew Reactions between Cyclobutanones and Alkenyl Groups

The use of saturated cyclobutanones provides opportunities to access various aliphatic ring systems. For example, β-branched cyclobutanones can undergo (4 + 2) cut-and-sew reactions to generate bridge bicycles. The seminal work by Murakami et al. demonstrated the synthesis of benzo-fused [3.2.1] bridged rings (Scheme 8),^9f^ though the scope is limited to the benzene linker due to the competing decarbonylation associated with the C–C activation of cyclobutanones. To address this challenge, in 2014 we found that the use of 2-aminopyridines^20^ as an additive can protect the cyclobutanone carbonyl from decarbonylation through the *in situ* imine formation and accelerate the reaction via acting as a temporary directing group (Scheme 9).^2^ Diverse [3.3.1] and [3.2.1] bridged rings with various linkers, such as nitrogen- or malonate-based ones, were constructed in good yields. These structures are challenging to access via the traditional type II intramolecular Diels–Alder (IMDA) reaction. Initial success with the enantioselective version of the reaction was also achieved (**30b**).

**Scheme 10:**
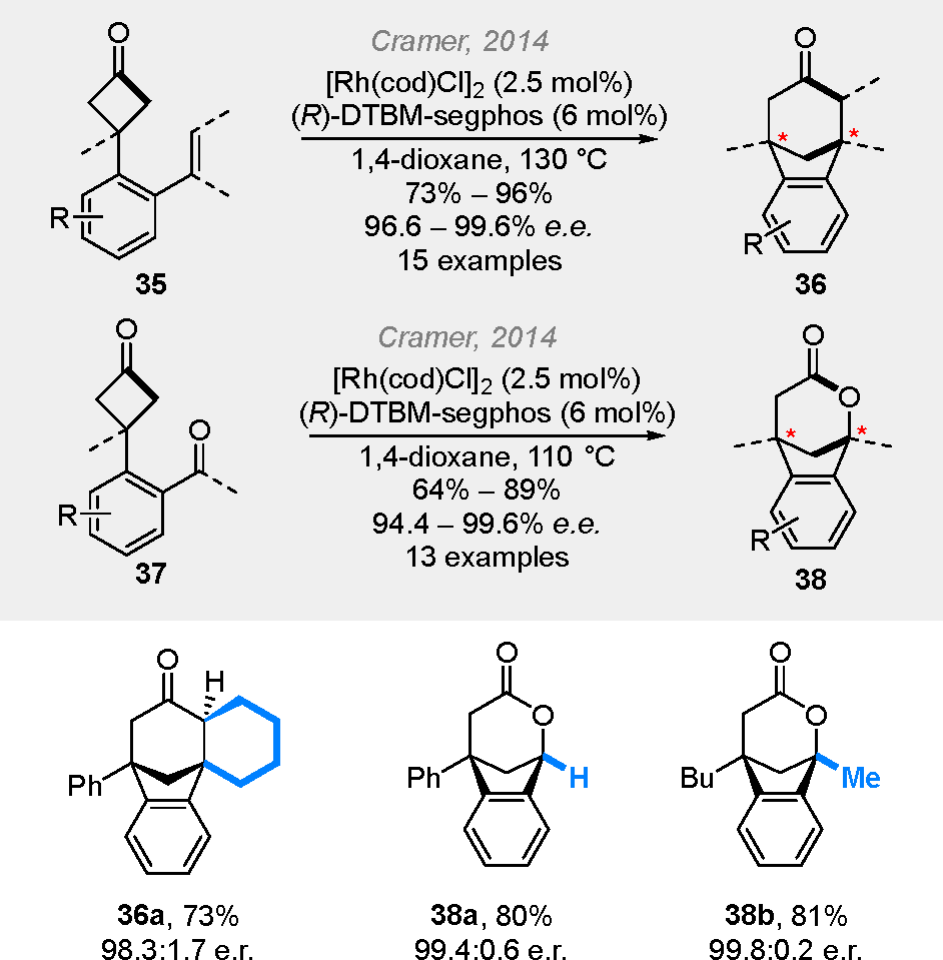
Rhodium-Catalyzed Cut-and-Sew Reactions between Cyclobutanones and Alkenyl and Carbonyl Groups

**Scheme 11:**
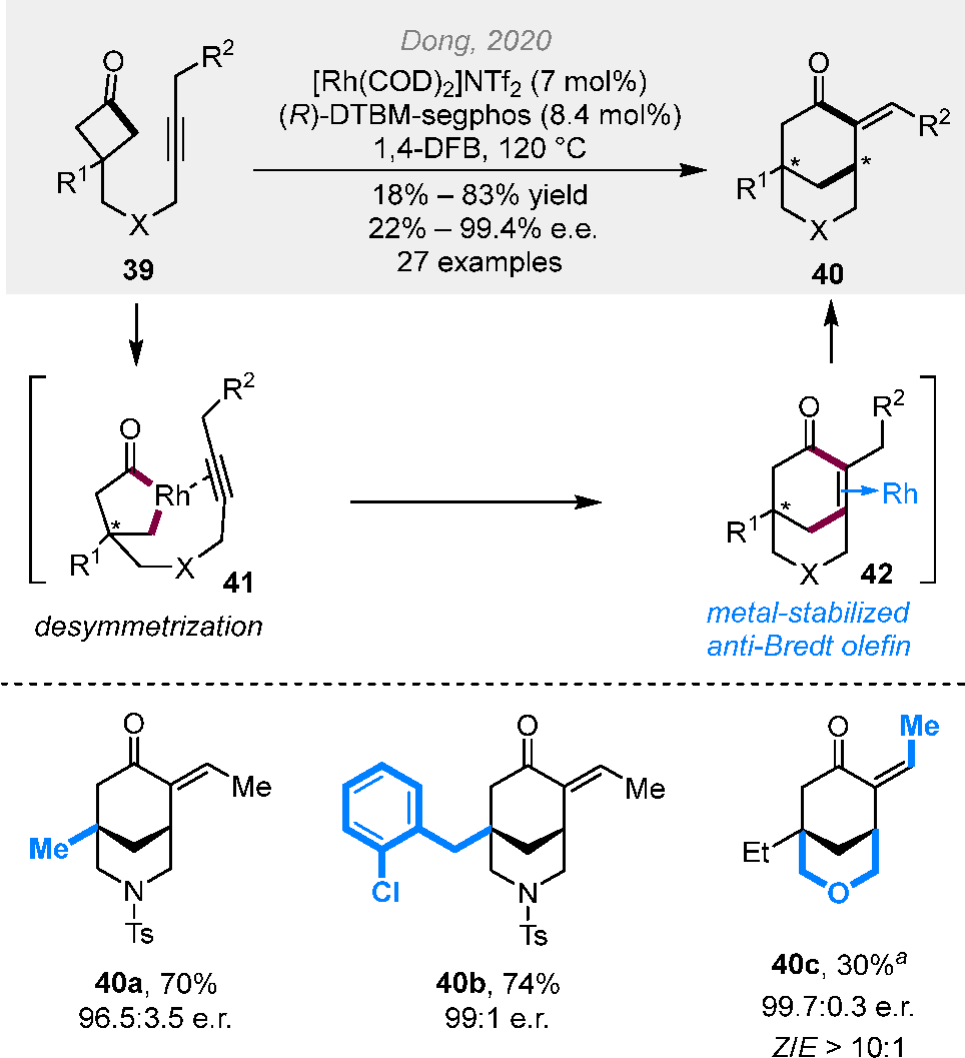
Rhodium-Catalyzed (4 + 2) Cut-and-Sew Reactions between Cyclobutanones and Alkynyl Groups [Rh(C_2_H_4_)_2_Cl]_2_ (5 mol %), AgSbF_6_ (10 mol %), (*R*)-DTBM-segphos (12 mol %), 1,4-dioxane, room temperature.

In 2014, the Cramer group reported the highly enantioselective (4 + 2) cut-and-sew reactions based on the benzene-tethered cyclobutanones using alkenyl and carbonyl groups as the coupling partners (Scheme 10).^21^ In these reactions, DTBM-segphos again proved to be the optional chiral ligand (vide supra, Scheme 3). In 2020, an enantioselective intramolecular cut-and-sew reaction between cyclobutanones and alkynyl groups was disclosed by us (Scheme 11).^22^ Excellent enantioselectivity was achieved using a cationic Rh-DTBM-segphos catalyst. Interestingly, the *in situ* generated anti-Bredt bridged rings can be stabilized by rhodium coordination, according to our DFT calculations, which then gives the olefin migration product through hydride transfer. The *E* alkene was found to be the thermodynamically favored product; in contrast, the kinetically favored *Z* product predominated with the oxygen-linked substrates (e.g., **40c**) when running the reaction at room temperature.

**Scheme 12:**
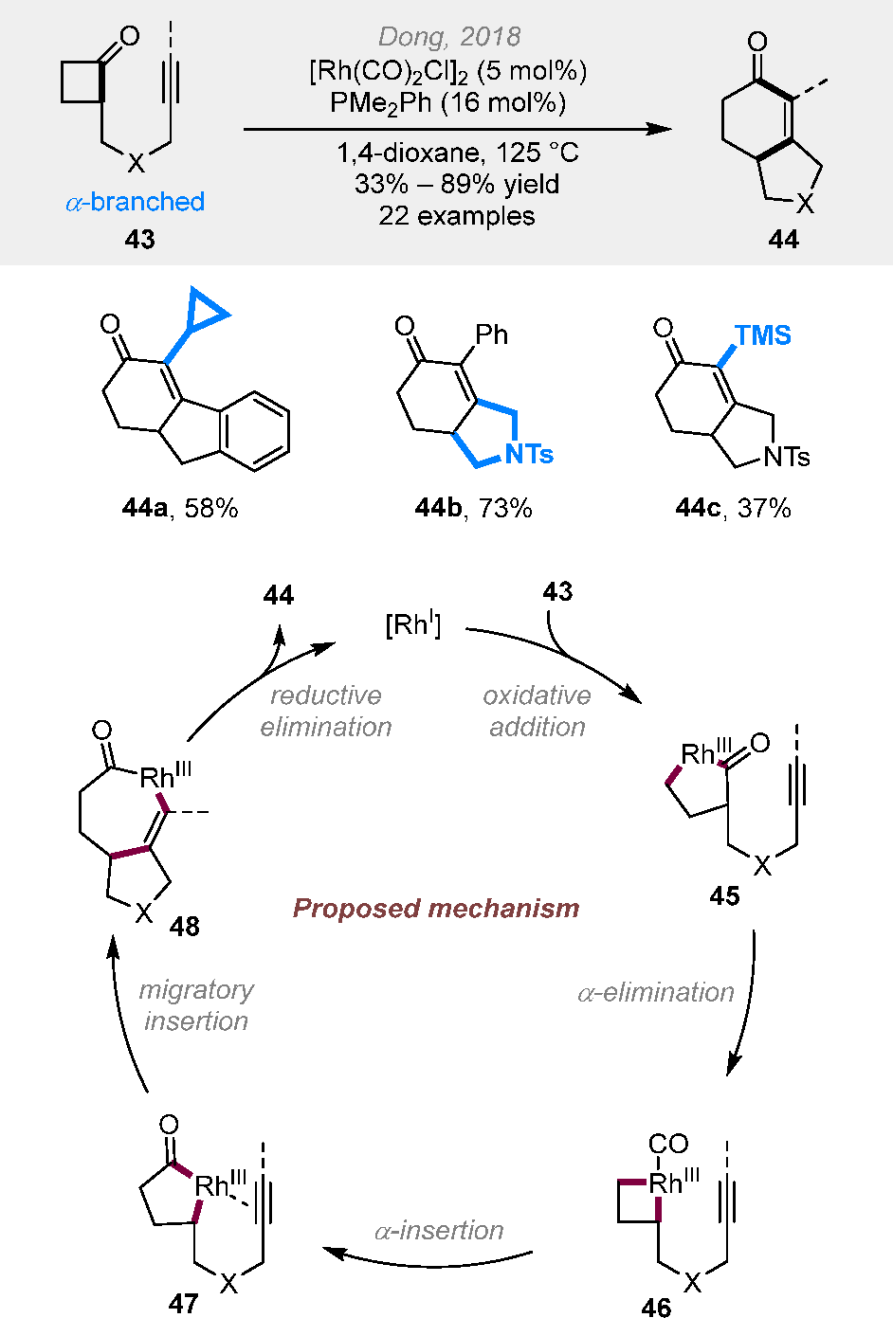
Rhodium-Catalyzed (4 + 2) Reactions between α-Branched Cyclobutanones and Alkynyl Groups

**Scheme 13:**
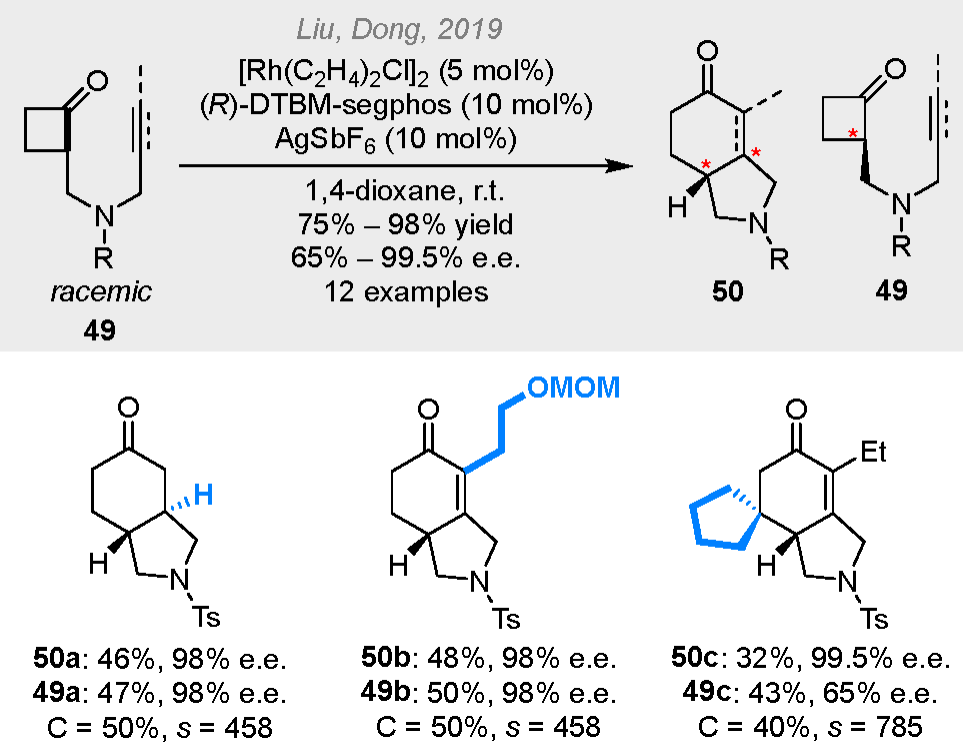
Kinetic Resolution of α-Branched Cyclobutanones via Fused-Ring Formation

In addition to the β-branched cyclobutanones, cyclobutanones with an unsaturated unit tethered to the α-position can lead to fused ring formation. In 2018, the cut-and-sew reaction between α-branched cyclobutanones and alkynyl groups was realized to construct [4.3.0] fused enones (Scheme 12).^23^ Electron-deficient [Rh(CO)_2_Cl]_2_ and less bulky, electron-rich PMe_2_Ph were found to be the optimal catalyst. Both carbon- and nitrogen-based linkers can be adopted; alkyl, aryl and silyl-substituted alkynyl groups are all suitable as coupling partners. Similar to the cut-and-sew reaction mechanism with benzocyclobutenones, it was proposed that the rhodium(I) catalyst first inserts into the less sterically hindered C–C bond in cyclobutanone **43** via oxidative addition to give complex **45**, followed by decarbonylation and CO-reinsertion to generate rhodacycle **47**. The sequential migratory insertion and reductive elimination finally deliver the fused-ring product **44**. Later, the combination of a cationic rhodium complex and DTBM-segphos ligand allowed an efficient kinetic resolution of α-branched cyclobutanones **49** via the selective fused-ring formation (Scheme 13).^24^ The reaction proceeded at room temperature with high *s* factors achieved. Under this condition, terminal alkenyl groups can also be coupled. The interesting role of DTBM-segphos in promoting favorable ligand-substrate dispersion interactions has been revealed by the DFT study.

### 2.3 (4 + 2 – 1) Cut-and-Sew Reactions of Benzocyclobutenones and Cyclobutanones

**Scheme 14:**
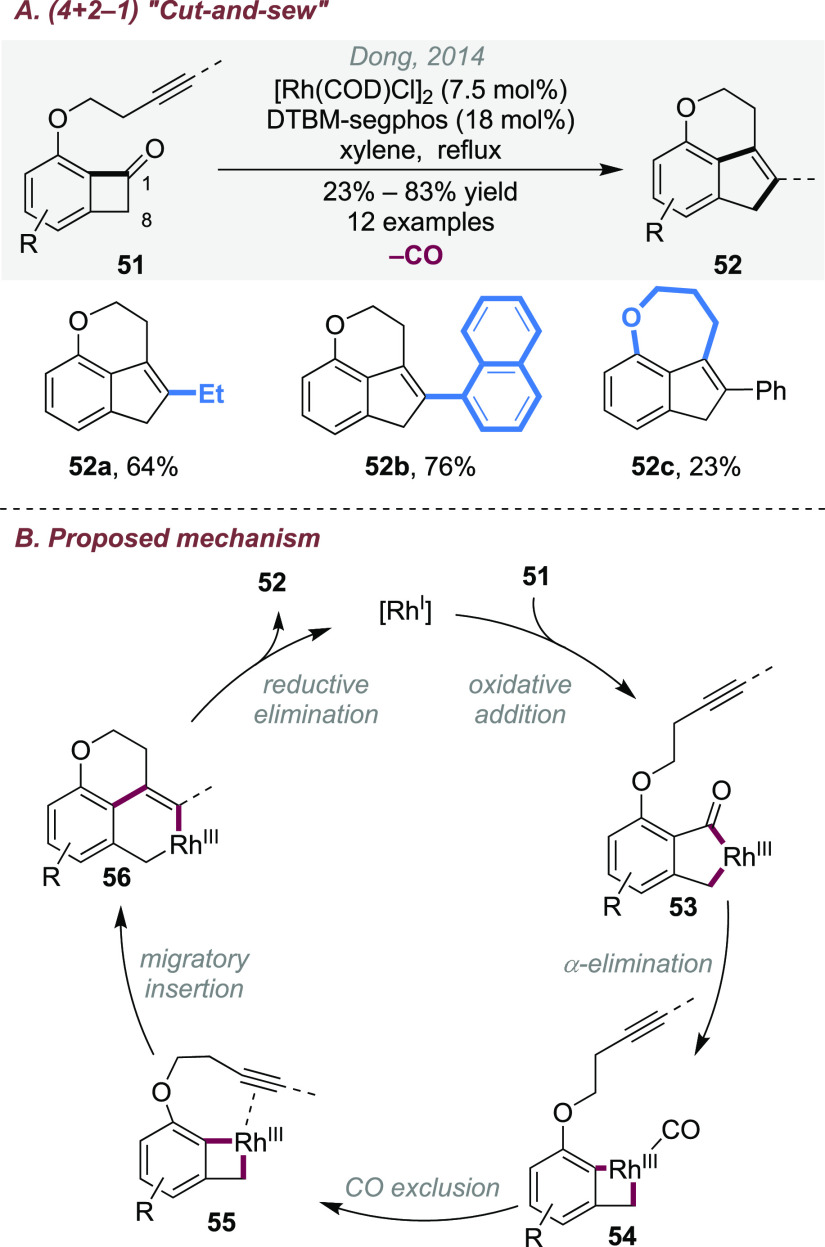
Rhodium-Catalyzed (4 + 2 – 1) Cut-and-Sew Reactions between Benzocyclobutenones and Alkynyl Groups

Considering that the carbonyl moiety in the substrates can be removed by decarbonylation, (4 + 2 – 1)-type transformations, namely, “decarbonylative cut-and-sew” reactions, have also been developed. This provides an unusual strategy to access bridged- or fused-ring scaffolds *without bearing a ketone moiety*. In 2014, the (4 + 2 – 1) reaction between benzocyclobutenones and alkynyl groups was discovered to give various fused indene products (Scheme 14A).^16^ The CO extrusion was promoted when running the reaction under reflux in xylene. It was proposed (Scheme 14B) that, after oxidation addition of rhodium(I) into the C1–C8 bond, decarbonylation and CO dissociation take place instead of reinsertion (vide supra, Scheme 2B).

**Scheme 15:**
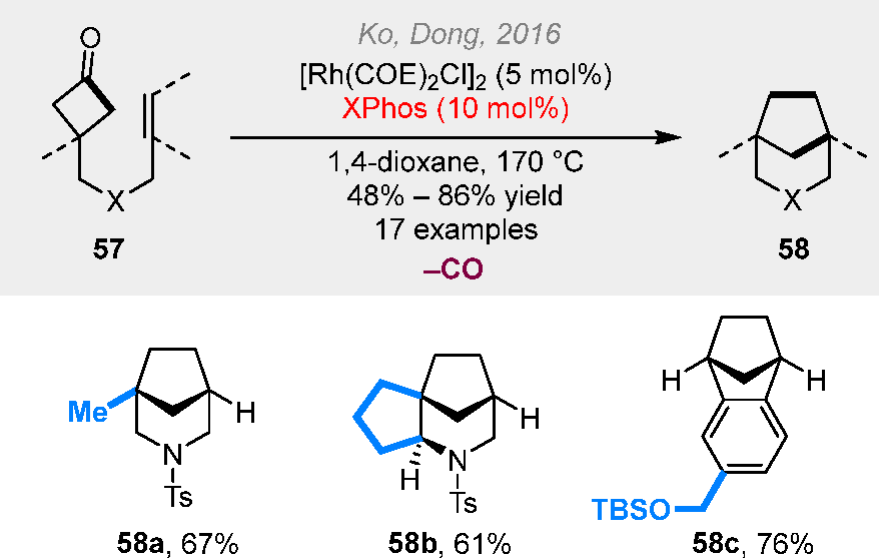
Rhodium-Catalyzed (4 + 2 – 1) Reactions between Cyclobutanones and Alkenyl Groups

The decarbonylative cut-and-sew reaction with cyclobutanones is more challenging, as it involves a difficult C(sp^3^)–C(sp^3^) reductive elimination. In 2016, we found that the use of a monodentate bulky Buchwald ligand enabled a smooth (4 + 2 – 1) cycloaddition between saturated cyclobutanones and alkenyl groups (Scheme 15).^25^ The bulkiness of the ligand not only promotes CO extrusion but also prevents the coordination of more than one phosphine ligand, thus resulting in coordinative unsaturation at the metal center for olefin coordination. Despite the high reaction temperature required for the reaction, we effectively obtained a range of cyclopentane-bridged rings that are otherwise challenging to prepare.

### 2.4 (4 + 1) Cut-and-Sew Reactions of Benzocyclobutenones and Cyclobutanones

**Scheme 16:**
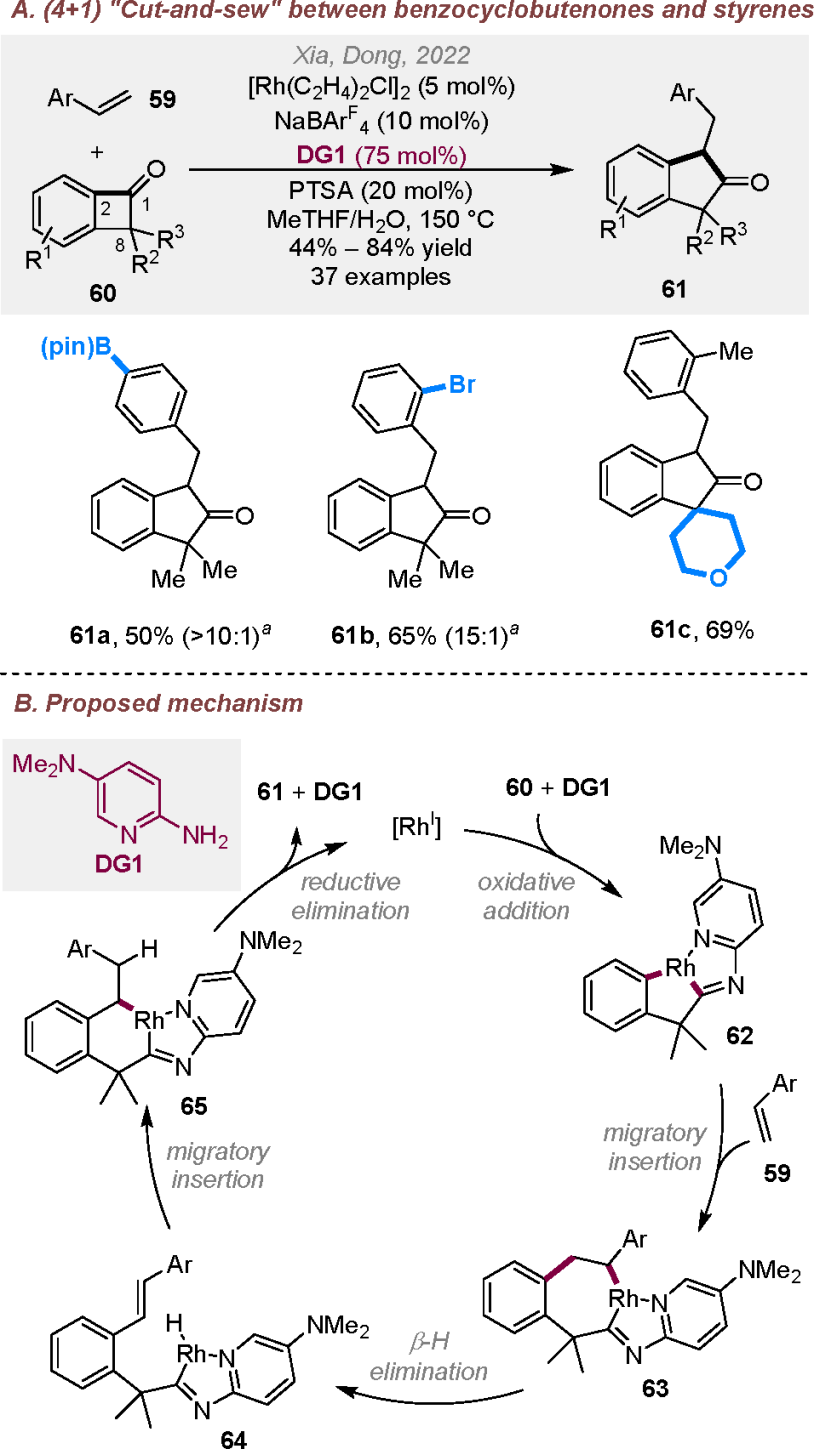
Rhodium-Catalyzed (4 + 1) Cut-and-Sew Reactions between Benzocyclobutenones and Styrenes Ratio of the (4 + 1) versus (4 + 2) products.

During our exploration of an intermolecular cut-and-sew reaction between benzocyclobutenones and styrenes, unexpected (4 + 1) cycloaddition products were obtained as the major products (Scheme 16A),^26^ in which the terminal carbon of styrenes inserted into the benzocyclobutenone C1–C2 bonds. The use of a “ligandless” cationic rhodium catalyst and a 2-aminopyridine additive were critical for this selectivity. While the double substitution at the C8 position of benzocyclobutenones is needed to prevent substrate decomposition, a range of multisubstituted 2-indanones was efficiently constructed by this method with good functional group tolerance. Further computational studies suggest that, rather than direct C–C reductive elimination, a competing β-H elimination takes place after C–C activation and 2π insertion, and the subsequent hydride reinsertion and reductive elimination lead to the five-membered ring formation (Scheme 16B).

**Scheme 17:**
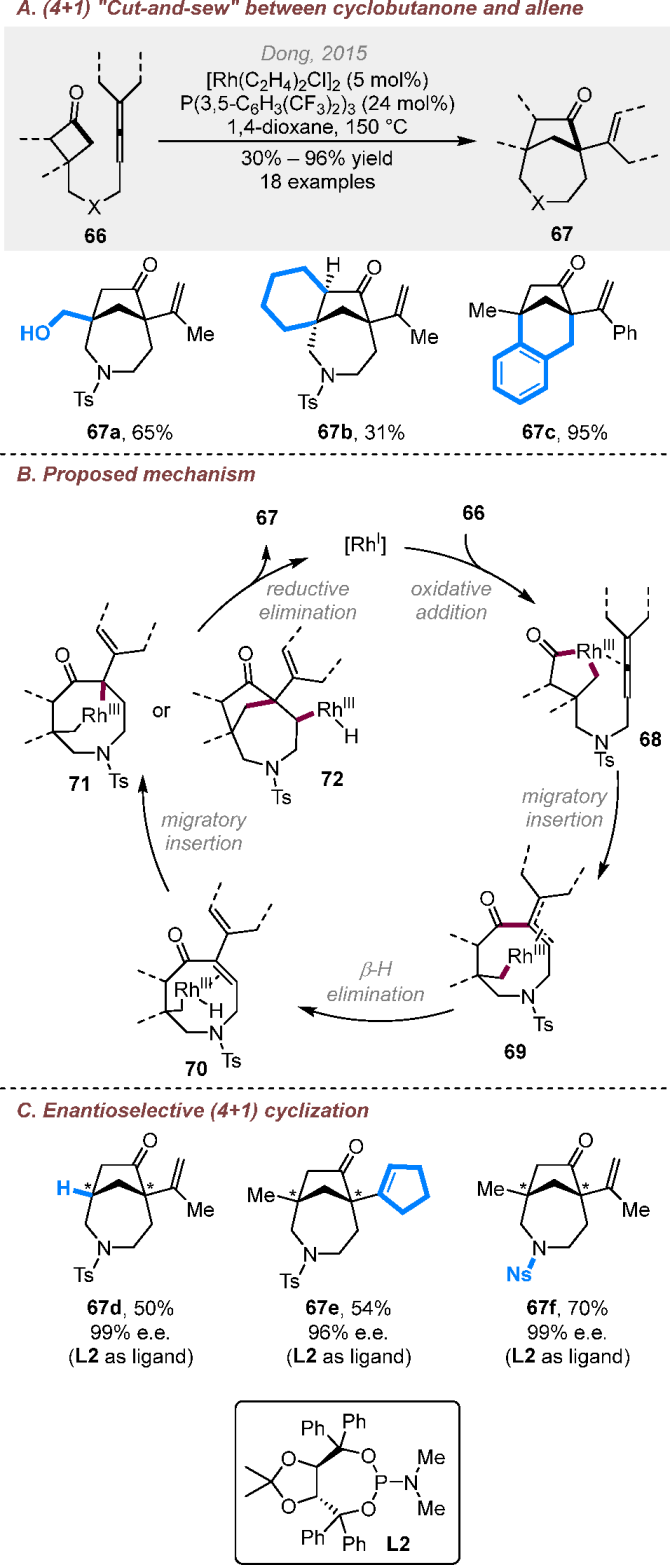
Rhodium-Catalyzed (4 + 1) Cut-and-Sew Reactions between Cyclobutanones and Allenyl Groups

In 2015, an intramolecular (4 + 1) cut-and-sew cycloaddition was discovered when using allene-tethered cyclobutanones as the substrates (Scheme 17A),^27^ which provides rapid access to [4.2.1] and [3.2.1] bicyclic structures. This method shows a good substrate scope on both the cyclobutanone and allene parts. On the basis of the deuterium labeling study, the reaction was proposed to start from the oxidation addition of Rh(I) into the α-C–C bond of cyclobutanone **66** to give intermediate **68**, in which the allene moiety coordinates to the rhodium center to prevent decarbonylation (Scheme 17B). The following migratory insertion of the acyl group into the allene central carbon generates allyl-rhodium complex **69**, which then undergoes β-H elimination to give diene **70**. At this stage, either C–H or C–C migratory insertion followed by reductive elimination can generate the bridged product. In addition, a highly enantioselective version of this reaction was realized using a TADDOL-derived phosphoramidite ligand (Scheme 17C).

## 3 Application in Total Synthesis

The [m.n.0] fused rings and [m.n.1] bridged rings obtained from the cut-and-sew reactions are often found in complex bioactive molecules; thus, a number of total syntheses based on such a deconstructive C–C activation strategy have been achieved.^28^ In this section, the synthetic efforts from our group are summarized.

### 3.1 Total Synthesis of Cycloinumakiol (Proposed Structure)

**Scheme 18:**
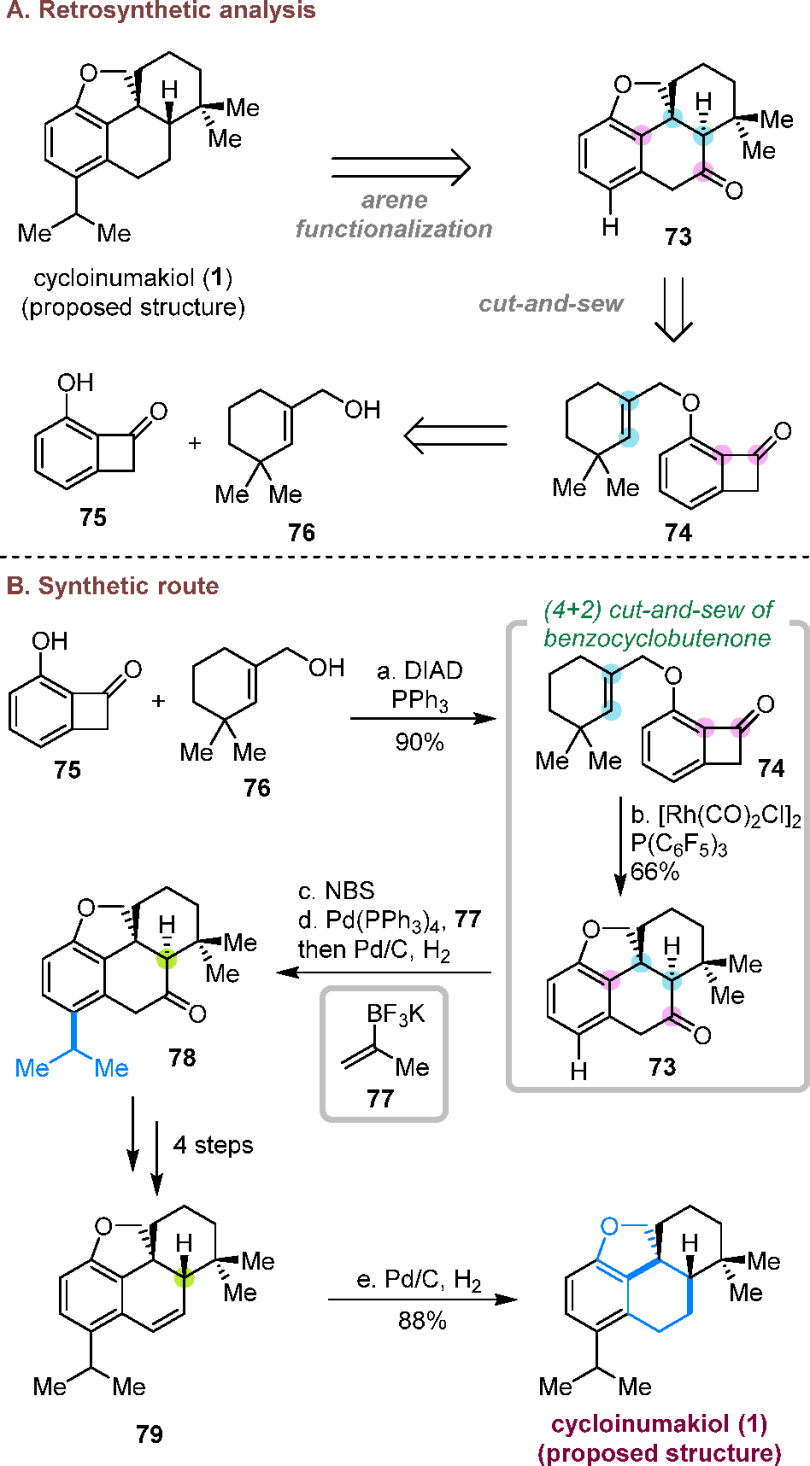
Total Synthesis of Cycloinumakiol (Proposed Structure)

Isolated from extracts of *Podocarpus latifolius*, cycloinumakiol (**1**) exhibits a distinct proposed chemical structure from other natural products in the tricyclic inumakiol family.^29^ In particular, it shows an unusual tetracyclic skeleton featuring a dihydrofuran ring and a quaternary carbon center. From a retrosynthetic aspect (Scheme 18A),^10a^ we envisioned that the isopropyl group on the phenyl ring could be introduced by late-stage arene functionalization, and the tetracyclic core could be constructed via the cut-and-sew reaction with a cyclohexene-tethered benzocyclobutenone. In a forward manner, the benzocyclobutenone substrate (**74**) was efficiently prepared from the Mitsunobu reaction between phenol **75** and alcohol **76**. To enable a highly challenging insertion of a sterically hindered trisubstituted alkenyl group, an electron-deficient [Rh(CO)_2_Cl]_2_/P(C_6_F_5_)_3_ catalyst was found to be optimal for this cut-and-sew reaction. With tetracycle **73** in hand, the isopropyl group was installed by a site-selective bromination and a one-pot Suzuki coupling/hydrogenation. After some end-game manipulations, the first total synthesis of the proposed structure of cycloinumakiol (**1**) was completed in nine steps from compounds **75** and **76**, whose structure was unambiguously determined by X-ray crystallography. This effort also let us elucidate the actual structure of cycloinumakiol, which was reassigned to 19-hydroxytotarol.

### 3.2 Asymmetric Total Synthesis of (−)-Cycloclavine

**Scheme 19:**
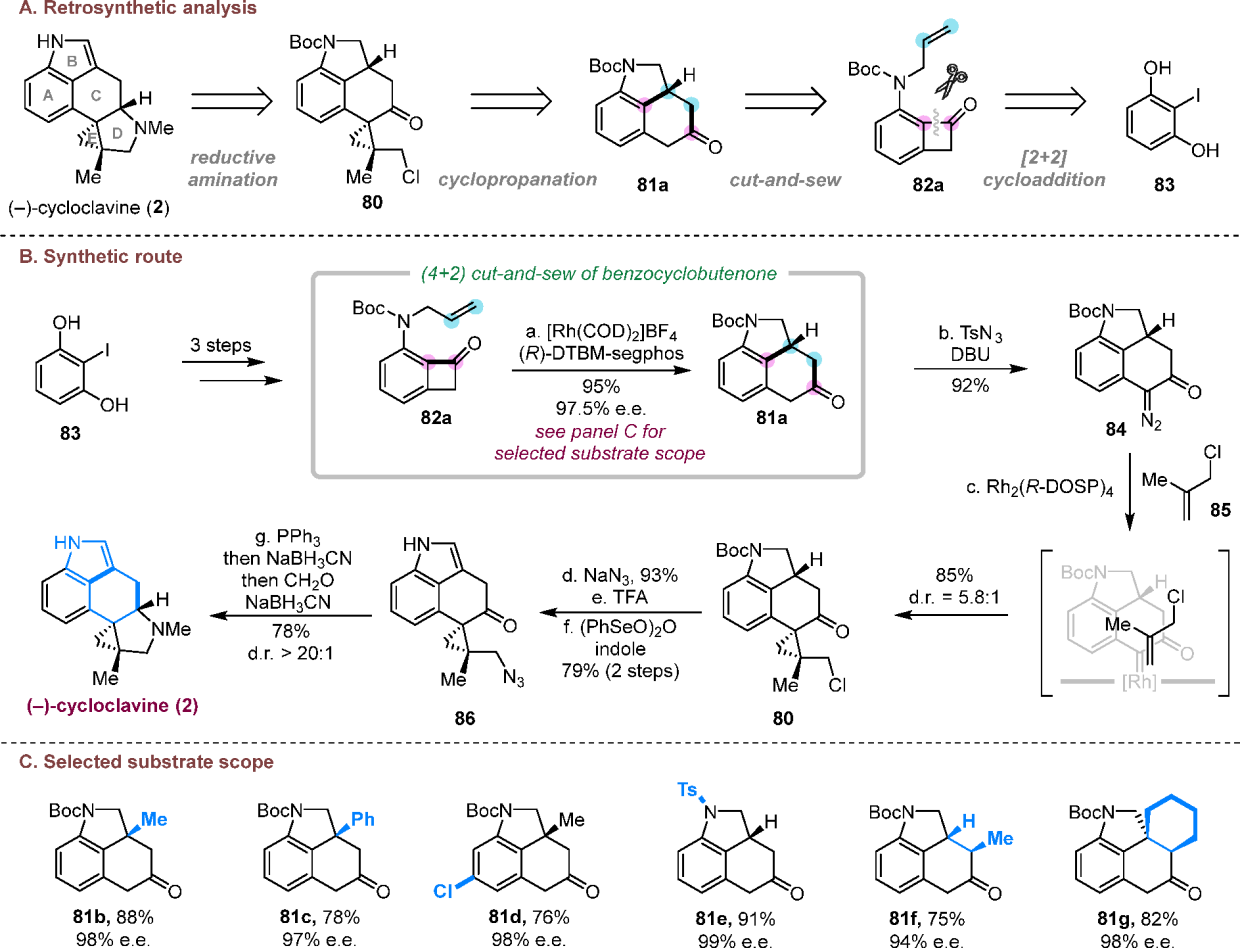
Enantioselective Total Synthesis of (−)-Cycloclavine

Isolated from the seeds of *Ipomoea hildebrandtii* by Hofmann and co-workers in 1969^30^ and later from *Aspergillus japonicas* in 1982,^31^ cycloclavine (**2**) is a unique member in the ergot alkaloid family because it processes a penta-cyclic core with a unique [3.1.0] structural motif. The sterically congested cyclopropane ring and three contiguous chiral centers including two adjacent quaternary carbons constitute additional challenges for the asymmetric total synthesis of cycloclavine. We envisioned a late-stage reductive amination tactic to construct the pyrrolidine D ring, a rhodium-catalyzed cyclopropanation to form the E ring, and an asymmetric cut-and-sew reaction to build the 6–6–5 fused A/B/C core structure (Scheme 19A).^3^ In a forward manner, the cut-and-sew precursor benzocyclobutenone **82a** was prepared in high yield from commercially available diphenol **83** in three steps (Scheme 19B). After detailed condition optimizations, the combination of cationic rhodium [Rh(COD)_2_]BF_4_ and DTBM-segphos was most efficient, giving the desired ketone **81a** in 95% yield and 97.5% e.e. This condition also appears to be quite general to access other nitrogen-containing tri- and tetracycles in high yields and excellent enantioselectivity (Scheme 19C). After the cut-and-sew step, the diazo-transfer followed by a Rh-catalyzed diastereoselective cyclopropanation^32^ of 2-methylallyl chloride **85** delivered the desired cyclopropane product **80** with good diastereoselectivity. In the end game, the S_N_2-substitution with azide, Boc deprotection and indoline oxidation gave indole **86**. A one-pot aza-Wittig/reduction/reductive amination delivered (−)-cycloclavine (**2**) in 78% yield and >20:1 diastereoselectivity. In summary, this C–C activation-based approach accomplished the asymmetric total synthesis of (−)-cycloclavine in only 10 steps with 30% overall yield.

### 3.3 Asymmetric Total Synthesis of (−)-Thebainone A

**Scheme 20:**
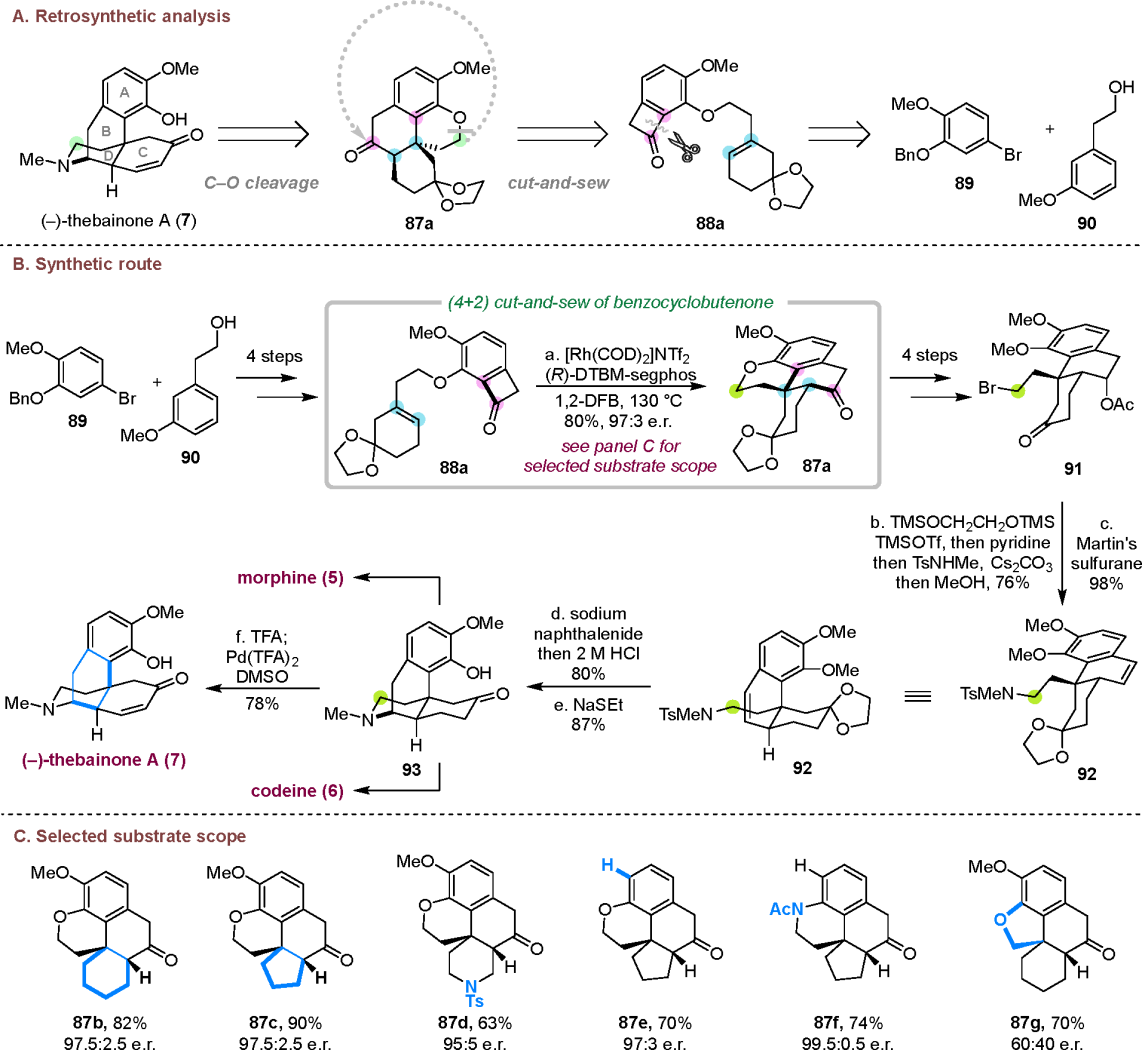
Deconstructive Asymmetric Total Synthesis of (−)-Thebainone A

Morphine (**5**) and its congeners are among the oldest and most studied alkaloid natural products, many of which have potent neurological and immunological activity.^33^ They generally possess a poly bridged/fused ring system, a quaternary center, a basic tertiary amine moiety and a 1,2,3,4-tetrasubstituted arene. As a unique member in the morphine-family alkaloids, thebainone A (**7**) contains an enone moiety in the C ring, which has served as a precursor to synthesize morphine (**5**) and codeine (**6**).^34^ However, the asymmetric synthesis of thebainone A was not reported previously. We devised a deconstructive strategy to synthesize (−)-thebainone A, in which the nitrogen-containing D ring is constructed in the end from an ether precursor (**87a**), and the fused A/B/C ring core structure, along with a quaternary carbon center, is assembled via an asymmetric cut-and-sew reaction (Scheme 20A).^10d^

In a forward manner, the cut-and-sew precursor **88a** was prepared in four steps from compounds **89** and **90** (Scheme 20B). The challenges of the key C–C activation-enabled (4 + 2) cycloaddition with compound **88a** were associated with the presence of an acid-sensitive ketal, a trisubstituted olefin coupling partner, and a relatively long linker. Ultimately, the use of [Rh(COD)_2_]NTf_2_ and DTBM-segphos as the catalyst combination afforded high yield and excellent e.e. with 1,2-difluorobenzene as the solvent. This condition can also be used to construct other tetracycles with different olefin coupling partners, linkers, and functional groups (Scheme 20C). With ketone **87a** in hand, the cyclic ether C–O bond was selectively cleaved by BBr_3_, and alkyl bromide **91** was accessed in four steps. Subsequently, ketone protection, S_N_2 amination, and Ac deprotection were achieved in one pot. The following dehydration with Martin’s sulfurane gave amine **92**. The piperidine D ring was constructed by a radical-mediated hydroamination of the alkenyl group through reduction of the tosylamide moiety. Finally, selective deprotection of the middle methyl ether by NaSEt gave ketone **93**, followed by desaturation by Stahl’s protocol,^35^ and furnished the first enantioselective total synthesis of (−)-thebainone A (**7**). It is worthy to note that intermediate **93** is also a known precursor to morphine (**5**) and codeine (**6**).^34,36^

### 3.4 Total Syntheses of Penicibilaenes A and B

**Scheme 21:**
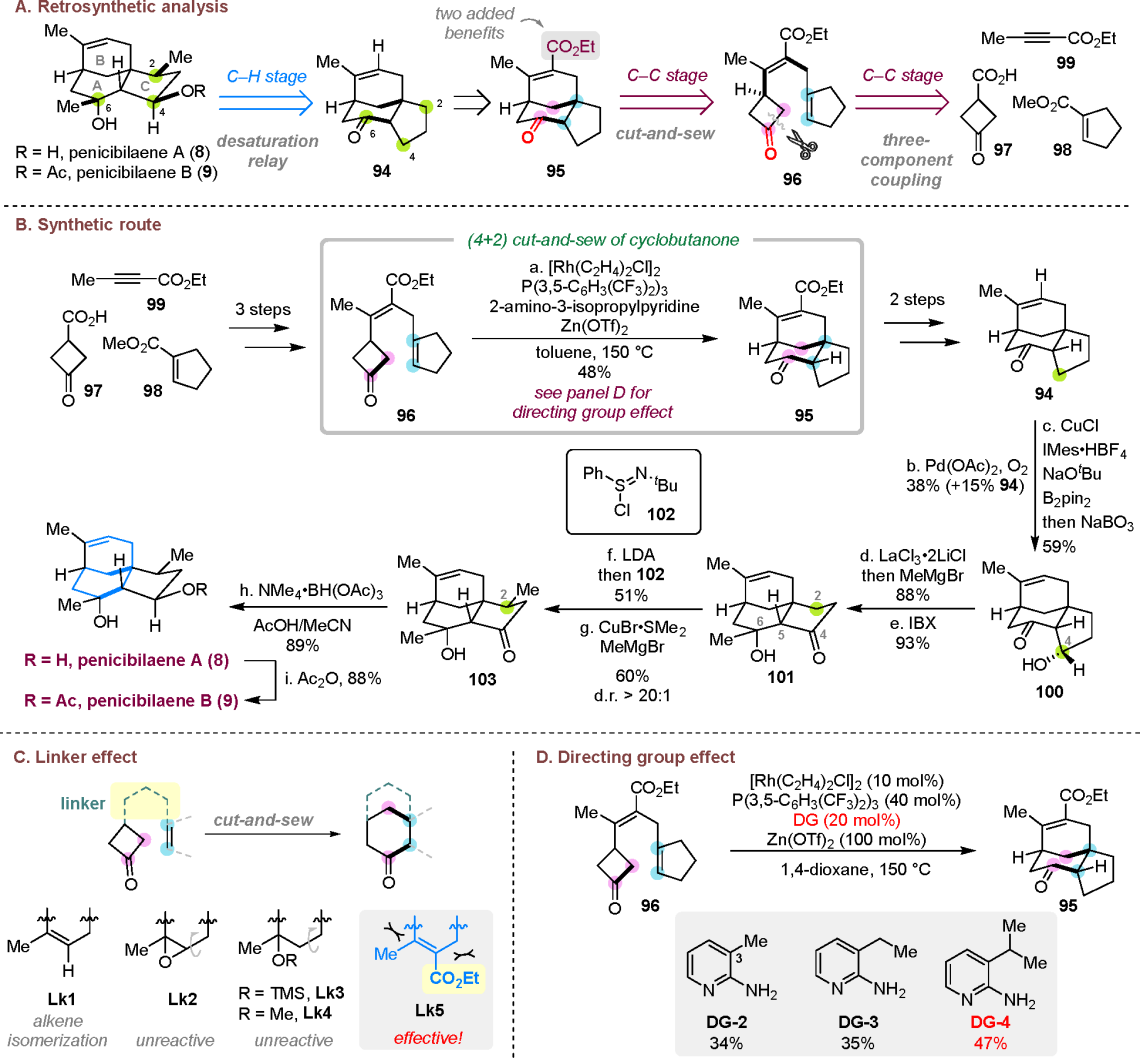
Total Syntheses of Penicibilaenes A and B

Isolated from marine fungus *Penicillium bilaiae* MA-267, penicibilaenes A (**8**) and B (**9**) are two sesquiterpenes showing selective and potent activity against plant pathogenic fungus *Colletotrichum gloeosporioides*.^37^ They possess an intriguing tricyclo[6.3.1.0^1,5^]dodecane skeleton, which is constituted by [3.3.1]-bridged and [4.3.0]-fused junctions. They also contain six chiral centers with five being contiguous and one all-carbon quaternary stereocenter. Inspired by the biomimetic “two-phase” approach for terpene synthesis,^38^ a “C–C/C–H” two-stage strategy was proposed to prepare penicibilaenes A and B (Scheme 21A).^4^ In the “C–H” stage, substituents and additional stereocenters are introduced to the core skeleton via sequential desaturation/β-functionalization of ketones, while in the “C–C” stage the carbon scaffold of the natural products is constructed by the cut-and-sew reaction.

In a forward style, the cut-and-sew precursor **96** was synthesized in three steps from commercially available starting materials **97**, **98**, and **99** (Scheme 21B). The key C–C activation reaction turned out to be nontrivial, and an important linker effect was found (Scheme 21C). Alkenyl (**Lk1**), epoxide (**Lk2**), silyl ether (**Lk3**), and methyl ether (**Lk4**) linkers were unsuccessful due to either their instability or lack of rigidity. Eventually, the ester-substituted alkenyl ester linker (**Lk5**) was most ideal because of its increased rigidity and stability; it also eased the substrate preparation. To address the challenge of coupling with a trisubstituted alkenyl group, 2-amino-3-isopropylpyridine (**DG-4**) was employed as the temporary carbonyl protecting group and directing group (Scheme 21D). It is also necessary to add zinc triflate to promote the cyclization, though its exact role remains to be uncovered. Ultimately, the desired cut-and-sew product (**95**) was obtained in useful yield, which then underwent decarboxylation to give tricycle **94**.

After constructing the core skeleton of the natural product in the “C–C” stage, the remaining functional groups and stereocenters were installed by taking advantage of a desaturation relay-based strategy involving consecutive ketone α,β-dehydrogenation and β-functionalization. A sequence of Stahl’s oxidation,^39^ conjugate boration^40^ and oxidation delivered β-hydroxyl ketone **100**. Chelation-controlled methylation^41^ followed by oxidation with IBX gave a new ketone (**101**) in high yield and diastereoselectivity. The methyl group at the C2 position was installed through another desaturation^42^/β-functionalization.^43^ The rigid half-cage scaffold allowed methyl addition from the less hindered convex face to deliver the desired diastereomer (**103**). Finally, an alcohol-directed Evans–Saksena reduction^44^ provided penicibilaene A (**8**) in 89% yield as a single diastereomer. Acylation of the less bulky secondary alcohol in penicibilaene A (**8**) delivered penicibilaene B (**9**). In summary, the first total syntheses of penicibilaenes A (**8**) and B (**9**) were accomplished in 13 and 14 steps, respectively, in the longest linear sequence from commercially available starting materials.

## 4 Summary and Outlook

In this Account, we have summarized our past efforts in the development of cut-and-sew reactions of benzocyclobutenones and cyclobutanones via catalytic C–C activation, as well as their applications in concise total synthesis of complex alkaloids and terpenoids. These methods prove to be useful for the construction of diverse bridged and fused rings that are otherwise more challenging to prepare. The reaction selectivity can be controlled by choices of the catalysts, ligands, and additives. Highly enantioselective cut-and-sew reactions have also been realized. Some of these transformations can even operate at room temperature.

As an outlook, a number of challenges and opportunities still exist for the further development of this type of transformation. First, the substrates so far are mainly restricted to highly strained rings. It would be more attractive if less strained, but readily available five- or six-membered ketones can undergo cut-and-sew reactions to construct medium-sized rings.^45^ In addition, the unsaturated coupling partners would benefit from a broader scope. For example, the coupling of tetra-substituted alkenyl groups remains to be achieved. Moreover, the type of linkers has large room to expand. To overcome the need of the Thorpe–Ingold effect, more active catalyst systems must be developed. Finally, most of these transformations are catalyzed by rhodium, and it could be an attractive direction to develop more practical and efficient first-row transition metal-catalyzed cut-and-sew reactions.

## References

[ref1] Xu T.; Dong G. Rhodium-Catalyzed Regioselective Carboacylation of Olefins: A C–C Bond Activation Approach for Accessing Fused-Ring Systems. Angew. Chem. Int. Ed 2012, 51, 7567–7571. 10.1002/anie.201202771. 22730207

[ref2] Ko H. M.; Dong G. Cooperative Activation of Cyclobutanones and Olefins Leads to Bridged Ring Systems by a Catalytic [4 + 2] Coupling. Nat. Chem. 2014, 6, 739–744. 10.1038/nchem.1989. 25054946 PMC4150356

[ref3] Deng L.; Chen M.; Dong G. Concise Synthesis of (−)-Cycloclavine and (−)-5-*epi*-Cycloclavine via Asymmetric C–C Activation. J. Am. Chem. Soc. 2018, 140, 9652–9658. 10.1021/jacs.8b05549. 29976068 PMC6677407

[ref4] Xue Y.; Dong G. Total Synthesis of Penicibilaenes via C–C Activation-Enabled Skeleton Deconstruction and Desaturation Relay-Mediated C–H Functionalization. J. Am. Chem. Soc. 2021, 143, 8272–8277. 10.1021/jacs.1c04335. 34038107 PMC9112325

[ref5] aLovering F.; Bikker J.; Humblet C. Escape from Flatland: Increasing Saturation as an Approach to Improving Clinical Success. J. Med. Chem. 2009, 52, 6752–6756. 10.1021/jm901241e. 19827778

[ref6] aNicolaou K. C.; Snyder S. A.; Montagnon T.; Vassilikogiannakis G. The Diels–Alder Reaction in Total Synthesis. Angew. Chem. Int. Ed 2002, 41, 1668–1698. 10.1002/1521-3773(20020517)41:10<1668::AID-ANIE1668>3.0.CO;2-Z. 19750686

[ref7] aMurakami M.; Ito Y. Cleavage of Carbon–Carbon Single Bonds by Transition Metals. Top. Organomet. Chem. 1999, 3, 97–129. 10.1007/3-540-68525-1_5.

[ref8] Chen P.-h.; Billett B. A.; Tsukamoto T.; Dong G. “Cut and Sew” Transformations via Transition-Metal-Catalyzed Carbon–Carbon Bond Activation. ACS Catal. 2017, 7, 1340–1360. 10.1021/acscatal.6b03210. 29062586 PMC5650109

[ref9] aHuffman M. A.; Liebeskind L. S.; Pennington W. T. Synthesis of Metallacyclopentenones by Insertion of Rhodium into Cyclobutenones. Organometallics 1990, 9, 2194–2196. 10.1021/om00158a009.

[ref10] aXu T.; Dong G. Coupling of Sterically Hindered Trisubstituted Olefins and Benzocyclobutenones by C–C Activation: Total Synthesis and Structural Revision of Cycloinumakiol. Angew. Chem. Int. Ed 2014, 53, 10733–10736. 10.1002/anie.201404802. PMC4214140 25138969

[ref11] aFlores-Gaspar A.; Martin R. Recent Advances in the Synthesis and Application of Benzocyclobutenones and Related Compounds. Synthesis 2013, 45, 563–580. 10.1055/s-0032-1316850.

[ref12] aAidhen I. S.; Ahuja J. R. A Novel Synthesis of Benzocyclobutenones. Tetrahedron Lett. 1992, 33, 5431–5432. 10.1016/S0040-4039(00)79113-1.

[ref13] Chen J.; Zhou Q.; Fang H.; Lu P. Dancing on Ropes - Enantioselective Functionalization of Preformed Four-Membered Carbocycles. Chin. J. Chem. 2022, 40, 1346–1358. 10.1002/cjoc.202100879.

[ref14] Lu G.; Fang C.; Xu T.; Dong G.; Liu P. Computational Study of Rh-Catalyzed Carboacylation of Olefins: Ligand-Promoted Rhodacycle Isomerization Enables Regioselective C–C Bond Functionalization of Benzocyclobutenones. J. Am. Chem. Soc. 2015, 137, 8274–8283. 10.1021/jacs.5b04691. 26051406 PMC4878673

[ref15] Xu T.; Ko H. M.; Savage N. A.; Dong G. Highly Enantioselective Rh-Catalyzed Carboacylation of Olefins: Efficient Syntheses of Chiral Poly-Fused Rings. J. Am. Chem. Soc. 2012, 134, 20005–20008. 10.1021/ja309978c. 23171396

[ref16] Chen P.-h.; Xu T.; Dong G. Divergent Syntheses of Fused β-Naphthol and Indene Scaffolds by Rhodium-Catalyzed Direct and Decarbonylative Alkyne–Benzocyclobutenone Couplings. Angew. Chem. Int. Ed 2014, 53, 1674–1678. 10.1002/anie.201310100. 24492973

[ref17] Zhu Z.; Li X.; Chen S.; Chen P.-h.; Billett B. A.; Huang Z.; Dong G. Cobalt-Catalyzed Intramolecular Alkyne/Benzocyclobutenone Coupling: C–C Bond Cleavage via a Tetrahedral Dicobalt Intermediate. ACS Catal. 2018, 8, 845–849. 10.1021/acscatal.7b03852. 29868245 PMC5983384

[ref18] Deng L.; Xu T.; Li H.; Dong G. Enantioselective Rh-Catalyzed Carboacylation of C=N Bonds via C–C Activation of Benzocyclobutenones. J. Am. Chem. Soc. 2016, 138, 369–374. 10.1021/jacs.5b11120. 26674855 PMC4884656

[ref19] Sun T.; Zhang Y.; Qiu B.; Wang Y.; Qin Y.; Dong G.; Xu T. Rhodium(I)-Catalyzed Carboacylation/Aromatization Cascade Initiated by Regioselective C–C Activation of Benzocyclobutenones. Angew. Chem. Int. Ed 2018, 57, 2859–2863. 10.1002/anie.201713179. 29360217

[ref20] Park Y. J.; Park J.-W.; Jun C.-H. Metal–Organic Cooperative Catalysis in C–H and C–C Bond Activation and Its Concurrent Recovery. Acc. Chem. Res. 2008, 41, 222–234. 10.1021/ar700133y. 18247521

[ref21] aSouillart L.; Parker E.; Cramer N. Highly Enantioselective Rhodium(I)-Catalyzed Activation of Enantiotopic Cyclobutanone C–C Bonds. Angew. Chem. Int. Ed 2014, 53, 3001–3005. 10.1002/anie.201311009. 24519918

[ref22] Hou S.-H.; Yu X.; Zhang R.; Deng L.; Zhang M.; Prichina A. Y.; Dong G. Enantioselective Type II Cycloaddition of Alkynes via C–C Activation of Cyclobutanones: Rapid and Asymmetric Construction of [3.3.1] Bridged Bicycles. J. Am. Chem. Soc. 2020, 142, 13180–13189. 10.1021/jacs.0c05647. 32619351 PMC8130001

[ref23] Deng L.; Jin L.; Dong G. Fused-Ring Formation by an Intramolecular “Cut-and-Sew” Reaction between Cyclobutanones and Alkynes. Angew. Chem. Int. Ed 2018, 57, 2702–2706. 10.1002/anie.201712487. PMC5849476 29338109

[ref24] Deng L.; Fu Y.; Lee S. Y.; Wang C.; Liu P.; Dong G. Kinetic Resolution via Rh-Catalyzed C–C Activation of Cyclobutanones at Room Temperature. J. Am. Chem. Soc. 2019, 141, 16260–16265. 10.1021/jacs.9b09344. 31568718 PMC7075347

[ref25] Zhou X.; Ko H. M.; Dong G. Synthesis of Bridged Cyclopentane Derivatives by Catalytic Decarbonylative Cycloaddition of Cyclobutanones and Olefins. Angew. Chem. Int. Ed 2016, 55, 13867–13871. 10.1002/anie.201608158. PMC5214575 27712025

[ref26] Ochi S.; Zhang Z.; Xia Y.; Dong G. Rhodium-Catalyzed (4 + 1) Cycloaddition between Benzocyclobutenones and Styrene-Type Alkenes. Angew. Chem. Int. Ed 2022, 61, e202202703 10.1002/anie.202202703. PMC9117520 35289979

[ref27] Zhou X.; Dong G. (4 + 1) vs (4 + 2): Catalytic Intramolecular Coupling between Cyclobutanones and Trisubstituted Allenes via C–C Activation. J. Am. Chem. Soc. 2015, 137, 13715–13721. 10.1021/jacs.5b09799. 26440740 PMC4884657

[ref28] aMurakami M.; Ishida N. Potential of Metal-Catalyzed C–C Single Bond Cleavage for Organic Synthesis. J. Am. Chem. Soc. 2016, 138, 13759–13769. 10.1021/jacs.6b01656. 27726343

[ref29] Devkota K. P.; Ratnayake R.; Colburn N. H.; Wilson J. A.; Henrich C. J.; McMahon J. B.; Beutler J. A. Inhibitors of the Oncogenic Transcription Factor AP-1 from *Podocarpus latifolius*. J. Nat. Prod 2011, 74, 374–377. 10.1021/np100736y. 21306129 PMC3064712

[ref30] Stauffacher D.; Niklaus P.; Tscherter H.; Weber H. P.; Hofmann A. Cycloclavin, Ein Neues Alkaloid aus *Ipomoea hildebrandtii* Vatke—71: Mutterkornalkaloide. Tetrahedron 1969, 25, 5879–5887. 10.1016/S0040-4020(01)83095-7. 5373534

[ref31] Furuta T.; Koike M.; Abe M. Isolation of Cycloclavine from the Culture Broth of *Aspergillus japonicus* SAITO. Agric. Biol. Chem. 1982, 46, 1921–1922. 10.1271/bbb1961.46.1921.

[ref32] Davies H. M. L.; Nagashima T.; Klino J. L. Stereoselectivity of Methyl Aryldiazoacetate Cyclopropanations of 1,1-Diarylethylene. Asymmetric Synthesis of a Cyclopropyl Analogue of Tamoxifen. Org. Lett. 2000, 2, 823–826. 10.1021/ol005563u. 10754686

[ref33] Blakemore P. R.; White J. D. Morphine, the Proteus of Organic Molecules. Chem. Commun. 2002, 1159–1168. 10.1039/b111551k. 12109065

[ref34] aGates M.; Tschudi G. The Synthesis of Morphine. J. Am. Chem. Soc. 1952, 74, 1109–1110. 10.1021/ja01124a538.

[ref35] Diao T.; Wadzinski T. J.; Stahl S. S. Direct aerobic α,β-Dehydrogenation of Aldehydes and Ketones with a Pd(TFA)_2_/4,5-Diazafluorenone Catalyst. Chem. Sci. 2012, 3, 887–891. 10.1039/C1SC00724F. 22690316 PMC3370690

[ref36] aWeller D. D.; Rapoport H. A Practical Synthesis of Codeine from Dihydrothebainone. J. Med. Chem. 1976, 19, 1171–1175. 10.1021/jm00232a001. 994145

[ref37] Meng L.-H.; Li X.-M.; Liu Y.; Wang B.-G. Penicibilaenes A and B, Sesquiterpenes with a Tricyclo[6.3.1.0^1,5^]dodecane Skeleton from the Marine Isolate of *Penicillium bilaiae* MA-267. Org. Lett. 2014, 16, 6052–6055. 10.1021/ol503046u. 25408229

[ref38] aChen K.; Baran P. S. Total Synthesis of Eudesmane Terpenes by Site-Selective C–H Oxidations. Nature 2009, 459, 824–828. 10.1038/nature08043. 19440196

[ref39] Diao T.; Stahl S. S. Synthesis of Cyclic Enones via Direct Palladium-Catalyzed Aerobic Dehydrogenation of Ketones. J. Am. Chem. Soc. 2011, 133, 14566–14569. 10.1021/ja206575j. 21851123 PMC3173566

[ref40] Lee K.-s.; Zhugralin A. R.; Hoveyda A. H. Efficient C–B Bond Formation Promoted by N-Heterocyclic Carbenes: Synthesis of Tertiary and Quaternary B-Substituted Carbons through Metal-Free Catalytic Boron Conjugate Additions to Cyclic and Acyclic α,β-Unsaturated Carbonyls. J. Am. Chem. Soc. 2009, 131, 7253–7255. 10.1021/ja902889s. 19432440 PMC2714532

[ref41] Krasovskiy A.; Kopp F.; Knochel P. Soluble Lanthanide Salts (LnCl_3_·2LiCl) for the Improved Addition of Organomagnesium Reagents to Carbonyl Compounds. Angew. Chem. Int. Ed 2006, 45, 497–500. 10.1002/anie.200502485. 16397856

[ref42] Mukaiyama T.; Matsuo J.-i.; Kitagawa H. A New and One-Pot Synthesis of α,β-Unsaturated Ketones by Dehydrogenation of Various Ketones with *N*-*tert*-Butyl Phenylsulfinimidoyl Chloride. Chem. Lett. 2000, 29, 1250–1251. 10.1246/cl.2000.1250.

[ref43] Cao M.-Y.; Ma B.-J.; Lao Z.-Q.; Wang H.; Wang J.; Liu J.; Xing K.; Huang Y.-H.; Gan K.-J.; Gao W.; Wang H.; Hong X.; Lu H.-H. Optically Active Flavaglines-Inspired Molecules by a Palladium-Catalyzed Decarboxylative Dearomative Asymmetric Allylic Alkylation. J. Am. Chem. Soc. 2020, 142, 12039–12045. 10.1021/jacs.0c05113. 32584568

[ref44] Evans D. A.; Chapman K. T.; Carreira E. M. Directed Reduction of *β*-Hydroxy Ketones Employing Tetramethylammonium Triacetoxyborohydride. J. Am. Chem. Soc. 1988, 110, 3560–3578. 10.1021/ja00219a035.

[ref45] aXia Y.; Ochi S.; Dong G. Two-Carbon Ring Expansion of 1-Indanones via Insertion of Ethylene into Carbon–Carbon Bonds. J. Am. Chem. Soc. 2019, 141, 13038–13042. 10.1021/jacs.9b07445. 31389237 PMC7075334

